# Somatic structural variation signatures in pediatric brain tumors

**DOI:** 10.1016/j.celrep.2023.113276

**Published:** 2023-10-17

**Authors:** Yang Yang, Lixing Yang

**Affiliations:** 1 Ben May Department for Cancer Research, University of Chicago, Chicago, IL 60637, USA; 2 Department of Human Genetics, University of Chicago, Chicago, IL 60637, USA; 3 University of Chicago Comprehensive Cancer Center, Chicago, IL 60637, USA; 4 Lead contact

## Abstract

Brain cancer is the leading cause of cancer-related death in children. Somatic structural variations (SVs), large-scale alterations in DNA, remain poorly understood in pediatric brain tumors. Here, we detect a total of 13,199 high-confidence somatic SVs in 744 whole-genome sequences of pediatric brain tumors from the Pediatric Brain Tumor Atlas. The somatic SV occurrences have tremendous diversity among the cohort and across different tumor types. We decompose mutational signatures of clustered complex SVs, non-clustered complex SVs, and simple SVs separately to infer their mutational mechanisms. Our finding of many tumor types carrying unique sets of SV signatures suggests that distinct molecular mechanisms shape genome instability in different tumor types. The patterns of somatic SV signatures in pediatric brain tumors are substantially different from those in adult cancers. The convergence of multiple SV signatures on several major cancer driver genes implies vital roles of somatic SVs in disease progression.

## In brief

Yang and Yang report 13,199 high-confidence somatic SVs from 744 pediatric brain tumors, revealing distinct molecular mechanisms shaping genome instability. Many SVs are functionally important in pediatric brain tumor progression.

## Graphical abstract

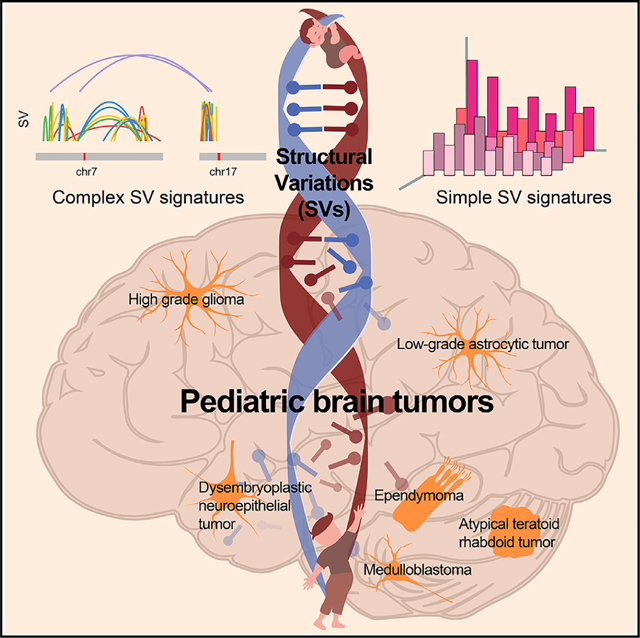

## INTRODUCTION

Brain and CNS cancers are the most prevalent solid tumors in children under 19 and the leading cause of cancer-related deaths among children.^1^ There are more than 100 types of pediatric brain tumors, which differ markedly from adult brain tumors.^2^ Although the 5-year survival rate of pediatric brain tumors is 75%, the survivors often suffer over their lifetimes from the effects of diseases and the side effects of treatments. Therefore, there is an urgent need to better understand the disease mechanisms and to develop new therapeutic strategies to further increase survival and improve the quality of life for patients and their families.

Genetic alterations in cancer include single-nucleotide variants (SNVs), copy number variants (CNVs), and structural variations (SVs). Pediatric brain tumors have few somatic SNVs but carry more somatic SVs than other pediatric cancers.^3^ SVs are large-scale structural changes of DNA, such as deletions, tandem duplications, inversions, and translocations. Some SVs can be quite complex; for example, chromothripsis refers to a single catastrophic event resulting in numerous SVs within one cell cycle.^4–6^ Understanding the mechanisms behind these alterations can not only improve our knowledge of disease biology but can also reveal therapeutic opportunities. For instance, translocations at the immunoglobulin gene locus in B cell lymphoma are caused by aberrant V(D)J recombination^7,8^ and often result in activation of *MYC* and *BCL2* oncogenes.^9,10^ Furthermore, breast and ovarian cancer patients carrying *BRCA1* and *BRCA2* mutations have a deficiency in DNA double-strand break repair and an elevated level of somatic SVs in their tumors.^11,12^ Patients with BRCA deficiency can be effectively treated by PARP inhibitors.^13,14^ Mutational signatures have been widely used to study the molecular mechanisms of SNVs,^15–17^ CNVs,^18,19^ SVs,^20^ and complex SVs^21^ in adult cancers. However, comprehensive studies of somatic SV signatures in pediatric brain tumors are still lacking. A recent study of SV signatures in pediatric high-grade gliomas has revealed that genetic alterations in the histone genes *TP53*, *CDKN2A*, and *RB1* are associated with complex SVs.^22^ However, whether other types of pediatric brain tumors harbor similar SV signatures remains unclear.

Here, we decompose complex and simple SV signatures from 744 pediatric brain tumors. We find tremendous heterogeneity in SV occurrences and SV signatures across tumor types.

## RESULTS AND DISCUSSION

### High-confidence somatic SVs in pediatric brain tumors

The Pediatric Brain Tumor Atlas (PBTA) has collected more than 1,000 pediatric brain tumors across more than 30 tumor types. There were 744 samples in the PBTA with whole-genome sequencing data after removing non-tumorous lesions, non-brain cancers, and non-primary cancer samples (Table S1). We focused on tumor types with at least 10 samples, including 220 low-grade astrocytic tumors (LGATs), 97 medulloblastomas, 71 ependymomas, 70 high-grade gliomas (HGGs), 44 gangliogliomas, 38 craniopharyngiomas, 27 atypical teratoid rhabdoid tumors (ATRTs), 23 meningiomas, 23 dysembryoplastic neuroepithelial tumors (DNETs), 17 non-meningothelial mesenchymal tumors, 13 schwannomas, 13 germ cell tumors, 13 neurofibromas, and 12 choroid plexus papillomas. Tumor types with fewer than 10 samples were classified as “Others.”

A previous study used Manta^23^ to detect somatic SVs in the PBTA cohort to study the effects of SVs on gene expression.^24^ However, the quality of variants called by a single algorithm is not always ideal.^25^ To produce high-confidence somatic SVs, we integrated three SV-calling algorithms: Meerkat,^26^ Manta, and Delly.^27^ Caller-specific SVs were discarded, and the somatic SVs detected by more than one algorithm were considered high confidence. Meerkat, Manta, and Delly detected 14,423, 55,934, and 9,475 somatic SVs in the 744 samples, respectively (Figure S1A). Because tumor DNA was not available, the SV quality could not be directly measured. Instead, we used CNV breakpoints detected by a read depth approach to assess the quality of SVs because a portion of somatic SVs change DNA copy numbers. We found that SVs detected by only one algorithm were not well supported by CNVs (Figure S1B), which suggested that caller-specific SVs had poorer quality. SVs detected by Manta with low read pair and split read support were of particularly poor quality (Figure S1C). SVs detected by more than one algorithm were better supported by CNVs (Figure S1B), which suggested that they were of high quality. We also removed deletions that resided at exon-intron boundaries that were likely caused by cDNA contamination.^25^

**Figure 1.**
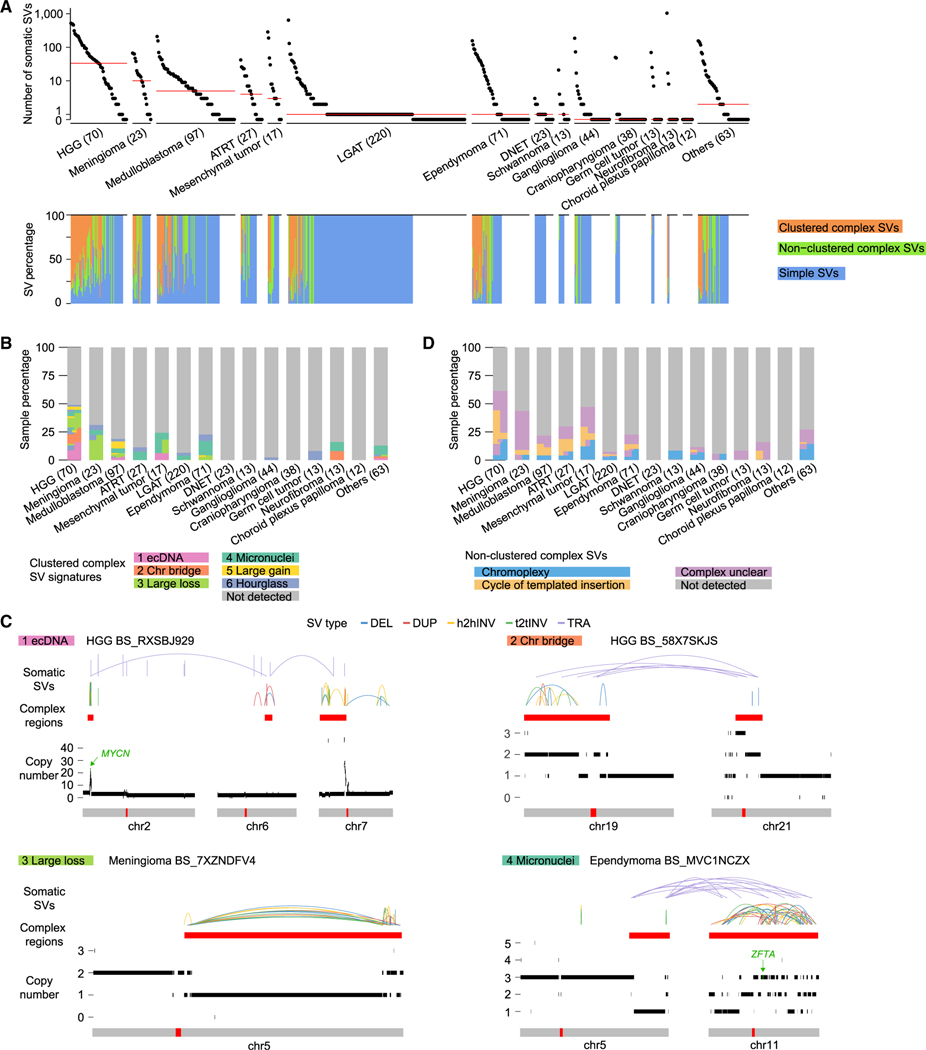
Somatic SVs and complex SVs in 744 pediatric brain tumors (A) Frequencies of somatic SVs and percentages of different types of SVs. Top: each dot represents one pediatric brain tumor sample. Samples are grouped by tumor type, and tumor types are sorted by median SV frequency (red lines) except for the “Others” category. The numbers in parentheses are sample sizes for the corresponding tumor types. Bottom: the percentages of clustered complex SVs, non-clustered complex SVs, and simple SVs in the corresponding samples at the top. HGG, high-grade glioma; ATRT, atypical teratoid/rhabdoid tumor; LGAT, low-grade astrocytic tumor; DNET, dysembryoplastic neuroepithelial tumor. (B and D) Percentages of clustered complex SV signatures (B) and percentages of non-clustered complex SVs (D). Each vertical block represents one tumor type, and each horizontal bar represents one sample. Samples are colored based on their SV signatures. Samples carrying multiple signatures have multiple colors arranged horizontally. The height of each sample may differ across tumor types depending on sample sizes of the tumor types. (C) Examples of clustered complex SVs. Colored arcs represent SVs of different types. The red bars below the colored arcs indicate regions of clustered complex SVs. Copy number profiles are displayed as black bars above the chromosome models. The red bars within the gray chromosome models indicate the locations of centromeres. Tumor types and sample IDs are shown next to the names of clustered complex SV signatures. DEL, deletion; DUP, tandem duplication; h2hINV, head-to-head inversion; t2tINV, tail-to-tail inversion; TRA, translocation. See also Figures S1–S3 and Table S2.

As a result, a total of 13,199 high-confidence SVs were detected from 744 pediatric brain tumors with a median of 3 SVs per sample. In each type of pediatric brain tumor, the number of somatic SVs per sample varied by nearly three orders of magnitude (Figure 1A). There was also considerable heterogeneity across tumor types. HGGs were most abundant in somatic SVs, followed by meningiomas and medulloblastomas, whereas no SVs were detected in choroid plexus papillomas (Figure 1A). Although most LGATs, ependymomas, gangliogliomas, and neurofibromas had very few SVs, a small fraction of them had very unstable genomes with more than 100 SVs (Figure 1A). In comparison, adult glioblastoma multiforme (GBM) and low-grade gliomas (LGGs) from the Pan-Cancer Analysis of Whole Genomes (PCAWG) cohort had medians of 98 and 7.5 somatic SVs, respectively (Figure S2A), which were more than for pediatric HGGs and LGATs. Note that, according to the World Health Organization (WHO) 2021 brain tumor classification, adult GBMs and pediatric HGGs are distinct tumor types. Although pediatric HGGs and adult GBMs originate from glial cells, the molecular and clinical characteristics are distinct.^28^ Furthermore, it is well known that malignant transformation of adult LGGs to GBMs is common, while transformation from pediatric LGATs to HGGs is rare.^29^ Pediatric LGATs are also distinct from adult LGGs.

### Complex SVs in pediatric brain tumors

A non-negative matrix factorization (NMF)-based approach has been very effective in decomposing mutational signatures for somatic SNVs^15–17^ and CNVs.^18,19^ Several studies,^20^ including the recent SV signature study in pediatric HGGs,^22^ also used this approach to extract SV signatures by combining complex SVs and simple SVs. Meaningful signatures can be reliably detected when DNA damage and repair mechanisms generate variants independently and repeatedly in cancer genomes. However, it is well established that chromothripsis events occur as one-time events, and the numbers of SVs vary extensively.^5,6,30^ Furthermore, multiple molecular mechanisms can lead to chromothripsis. For example, lagging chromosomes trapped in micronuclei during mitosis can shatter into many pieces, and some fragments can be ligated together in chromothripsis events.^5^ Dicentric chromosomes can form chromatin bridges during cell division, shatter into pieces, and also produce chromothripsis.^6^ NMF-based SV signature decomposition cannot differentiate these mechanisms. To better deduce SV signatures in pediatric brain tumors, we studied clustered complex SVs, non-clustered complex SVs, and simple SVs separately. Clustered complex SVs, like chromothripsis, are those with breakpoints enriched in certain genomic regions. Circular extrachromosomal DNA (ecDNA) with many SV breakpoints is also a clustered complex SV.^21^ We recently developed Starfish, a clustering-based approach, to infer clustered complex SV signatures based on their SV and CNV patterns.^21^ We reported six clustered complex SV signatures using nearly 2,500 adult tumors, including micronucleus-induced chromothripsis, chromatin-bridge-induced chromothripsis, and ecDNA. There are three other signatures that cannot be linked to biological processes; namely, “Large loss,” “Large gain,” and “Hourglass.” Non-clustered complex SVs are complex SVs with scattered breakpoints, including chromoplexy and cycle of templated insertions.^20^ Chromoplexy events are likely formed through the repair of multiple co-occurring DNA double-strand breaks similar to reciprocal translocations,^31,32^ whereas templated insertions may reflect replication-based mechanisms.^20,26,33^ After detecting clustered and non-clustered complex SVs, the remainder of SVs were classified as simple SVs, which include deletions, tandem duplications, balanced/unbalanced/foldback inversions, and balanced/unbalanced translocations.

Among the 13,199 SVs in 744 pediatric brain tumors, 7,601 (57.6%) were clustered complex SVs that belonged to 146 individual complex events, 2,377 (18.0%) were non-clustered complex SVs that belonged to 346 events, and 3,221 (24.4%) were simple SVs (Table S2). Of the 744 tumors, 108 (14.5%) and 150 (20.2%) carried clustered and non-clustered complex SVs, respectively, whereas 552 (74.2%) did not have any complex SVs. The high numbers of SVs in tumors with very unstable genomes (>100 SVs) mainly arose from complex SVs (Figure 1A; Figure S3A). HGGs had the highest abundance of complex SVs, whereas DNETs did not carry any complex SVs (Figure 1A). We used Starfish^21^ to classify clustered complex SV signatures (Table S3) and used a junction pattern^20^ to determine non-clustered complex SVs. HGGs and medulloblastomas^22^ carried nearly all types of complex SV signatures, whereas other tumor types only harbored a few types of complex SV signatures (Figure 1B). Chromatin-bridge-induced chromothripsis (“Chr bridge” signature) (Figure 1C) only occurred in HGGs and neurofibromas (Figure 1B). Hourglass chromothripsis events (“Hourglass” signature), complex SVs with a small amount of DNA loss and highly concentrated SV breakpoints, were detected in a small number of samples in several tumor types (Figure 1B), such as HGGs, meningiomas, medulloblastomas, and ependymomas. The “ecDNA” (Figure 1C) signature was predominantly found in HGGs. The “Large loss” signature (Figure 1C), characterized by complex SVs with a large amount of DNA loss, was mainly observed in HGGs and meningiomas. Micronucleus-induced chromothripsis (“Micronuclei” signature) (Figure 1C) was enriched in mesenchymal tumors and ependymomas (Figure 1B). We note that Starfish does not differentiate the circular form of ecDNA and the linear form of the homogenously staining region (HSR). Because the “ecDNA” signature was primarily detected in HGGs, and ecDNA has been reported in HGGs frequently leading to amplifications of *MYCN*,^22^ we refer to this signature as “ecDNA” in this manuscript. Our “ecDNA” signature is a clustered complex SV signature and is used to differentiate these events from other types of complex SVs, such as micronucleus-induced chromothripsis. Besides the complex form, there is also a simple form of ecDNA, which is not captured by this signature. Regarding non-clustered complex SVs, chromoplexy was found in many different tumor types and occurred in as many as 18.6% (13 of 70) of HGGs and 17.6% (3 of 17) of mesenchymal tumors (Figure 1D). Cycle of templated insertions was abundant in HGGs (Figure 1D). However, adult GBMs were more abundant in complex SVs than pediatric HGGs (Figures S2A–S2C), with ecDNA being the most common clustered complex SV (Figure S2B).

In summary, different types of pediatric brain tumors often carry distinct complex SV signatures.

### Simple SVs in pediatric brain tumors

**Figure 2.**
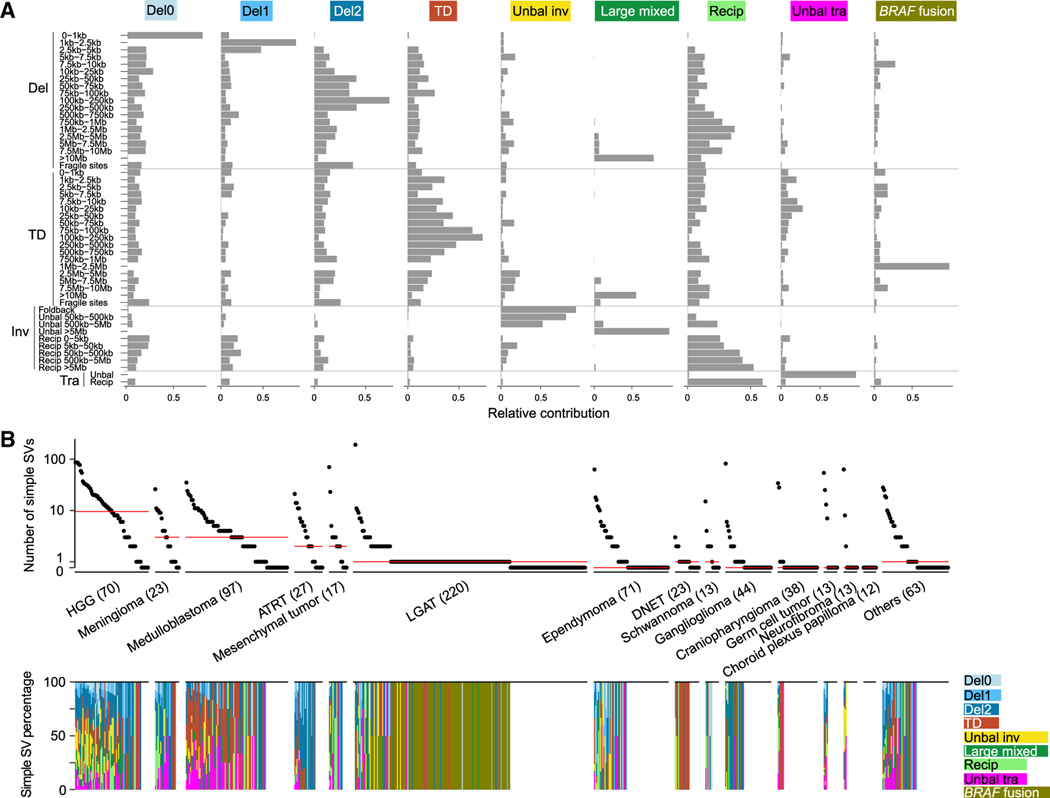
Simple SV signatures and their distributions (A) Nine simple SV signatures of 744 pediatric brain tumors. The four major SV categories and 49 subcategories of simple SVs are shown on the y axis. The names of the nine simple SV signatures are displayed at the top. The relative contributions of SV subcategories to the corresponding signatures are shown on the x axis. (B) Frequencies of simple SVs and percentages of simple SV signatures. Top: each dot represents one sample. Samples are grouped by tumor types. Red bars indicate median frequencies. The numbers in parentheses are sample sizes for the corresponding tumor types. Bottom: the percentages of simple SV signatures in the corresponding samples at the top. See also Figures S2 and S3.

Next, we used an NMF-based algorithm, SigProfilerExtractor,^34^ to decompose simple SV signatures; a total of nine signatures were extracted (Figure 2A; Table S2). We further decomposed simple SV signatures using another algorithm, signeR,^35^ and found very similar signatures (Figure S3B). We will use signatures decomposed by SigProfilerExtractor in the remainder of this manuscript. The simple SV signatures included deletions smaller than 1 kb (“Del0”), deletions between 1 and 5 kb (“Del1”), deletions larger than 5 kb and shorter than 10 Mb (“Del2”), shorter than 10 Mb tandem duplications (“TDs”), unbalanced inversions (“Unbal inv”), large intra-chromosomal SVs (“Large mixed”), reciprocal inversions and reciprocal translocations (“Recip”), as well as unbalanced translocations (“Unbal tra”). Interestingly, large TDs resulting in *KIAA1549*-*BRAF* fusions belonged to a standalone signature; namely, “*BRAF* fusion.” *KIAA1549*-*BRAF* fusion is known to be the most frequent genetic alteration in LGATs.^36–38^ HGGs had more simple SVs than other tumor types, while a considerable number of samples from various tumor types, including LGATs and ependymomas, showed no evidence of any SVs, including simple SVs (Figure 2B). Most tumors exhibiting simple SVs carried several distinct simple SV signatures (Figure 2B), and apparent enrichments could be observed. For example, DNETs predominantly carried the “TD” signature, schwannomas mainly harbored the “Recip” signature, the “Unbal inv” signature was mainly found in HGGs, the “TD” signature was enriched in medulloblastomas, the “Del2” signature was abundant in ATRTs, and the “*BRAF* fusion” signature was almost exclusive to LGATs (Figure 2B). Intriguingly, 102 of 220 LGATs had *KIAA1549*-*BRAF* fusions, and among the fusion-positive LGATs, 88 had the fusion as the only SV present in their genomes. These results suggested that the mutational mechanism leading to the TDs and fusions is not very active in LGAT precursor cells because this mechanism does not repeatedly produce somatic SVs in LGATs. Because LGATs with *BRAF* fusions have early disease onset (SVs associated with clinical properties), and no other SVs are generated except the ones at the *BRAF* locus, it is also possible that the fusion may suppress SV formation. In our recent study on simple SV signatures in adult cancers, a total of 13 simple SV signatures were decomposed.^39^ No dominant simple SV signatures were observed in adult GBMs and LGGs (Figure S2D).

Taken together, our complex SV and simple SV signature analysis demonstrated that numerous mutational mechanisms are active in pediatric brain tumors to induce genome instability, with unique molecular mechanisms present in different tumor types.

### Genomic features associated with SV signatures

**Figure 3.**
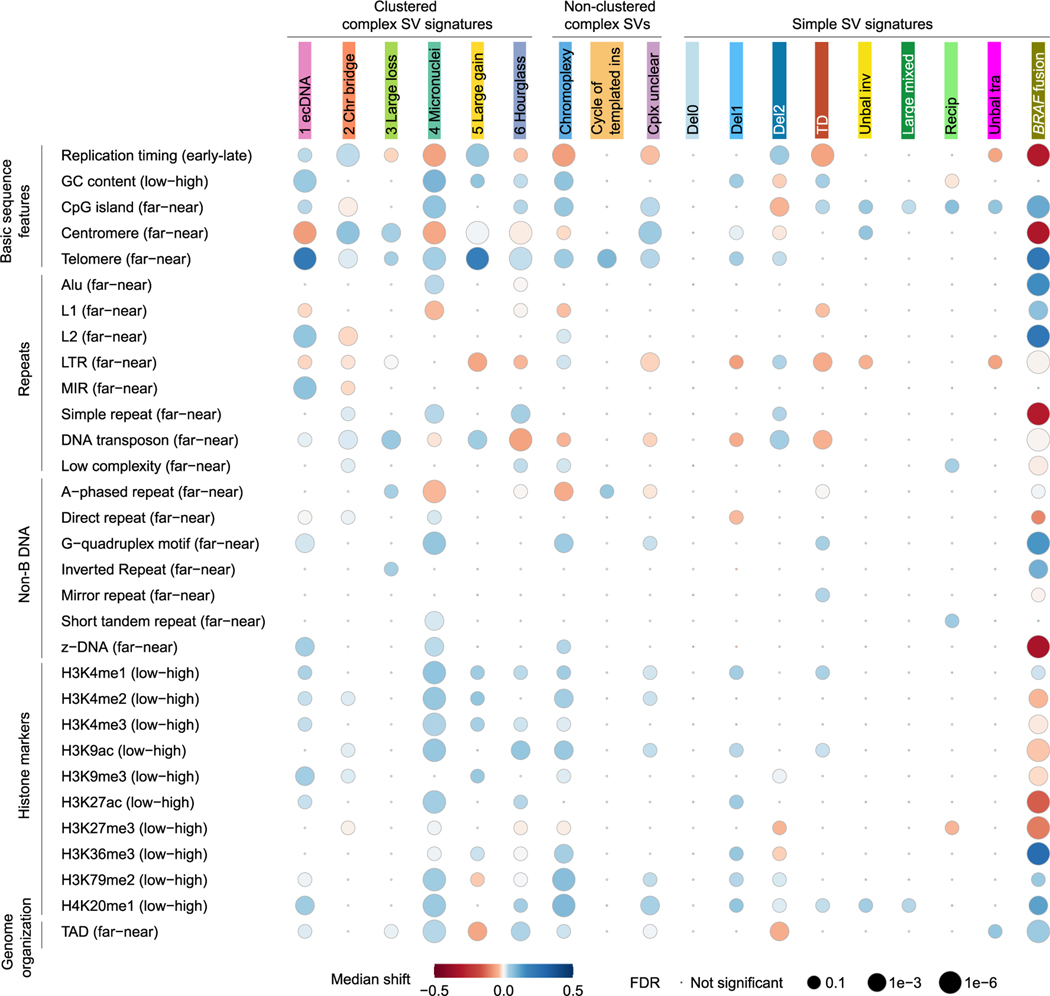
Associations of SV signatures with 31 genomic features SV signatures and genomic features are listed on the x and y axes, respectively. Each dot represents the association between one SV signature and one genomic feature. The size of dots reflects significance levels. The colors of the dots indicate the direction of the median shift of the given signature relative to the given feature in terms of the extremes of the parameters listed in parentheses next to each feature; red indicates a shift toward the extreme listed on the left in parentheses (e.g., early, low, far, etc.), and blue indicates a shift toward the extreme listed on the right in parentheses (e.g., late, high, near, etc.). For instance, the dot for the “ecDNA” signature association with the centromere feature is colored red, indicating that the observed SV breakpoints of this signature are farther away (left in the parentheses) from centromeres than randomized breakpoints. The red/blue colors of this figure representing directions of biases are the same as in Figure 5C of Li et al.^20^ (simple SV biases in adult cancers) and Figure 5A of Bao et al.^21^ (clustered complex SV biases in adult cancers). See also Figures S4 and S5.

Somatic SVs are not evenly distributed across the genome.^20^ Many factors, such as replication timing, GC content, repeat content, and 3D genome organization, have been associated with SV breakpoint distribution.^20,40^ Here, we surveyed 31 genomic features for their relationships with somatic SVs in pediatric brain tumors (Figure 3). The breakpoint biases were calculated in the same manner as in Li et al.^20^ and Bao et al.^21^

For clustered complex SV signatures, SV breakpoints of the “ecDNA,” “Chr bridge,” and “Large gain” signatures were significantly enriched in late-replicated regions (Figure 3; Figure S4). In contrast, all clustered complex SV signatures in adult cancers were enriched in early-replicated regions.^21^ In adult cancers, all clustered complex SV signatures were enriched in GC-rich regions and near CpG islands,^21^ whereas only the “ecDNA” and “Micronuclei” signatures in pediatric brain tumors were enriched in GC-rich regions (Figure 3; Figure S4). SV breakpoints of the “Large loss” signature were significantly closer to centromeres than expected in pediatric brain tumors (Figure 3; Figure S4), in a pattern opposite to adult cancers.^21^ In adult cancers, the “ecDNA” and “Chr bridge” signatures were significantly farther away from telomeres, whereas other clustered complex SVs were significantly closer to telomeres.^21^ However, all clustered complex SV signatures were significantly closer to telomeres in pediatric brain tumors (Figure 3; Figure S4). All clustered complex SV signatures in adult cancers were significantly closer to many types of repetitive elements (simple repeats, short tandem repeats, and transposable elements),^21^ whereas in pediatric brain tumors, the repetitive elements had variable effects. For example, the “Micronuclei” signature in pediatric brain tumors was enriched near Alu elements but depleted around L1s (Figure 3). Therefore, the SV breakpoint distributions of clustered complex SVs in pediatric brain tumors were quite different from those of adult cancers.

Chromoplexy has been proposed to form through simultaneous ligation of multiple broken chromosomal ends, similar to reciprocal translocations.^31,32^ In adult cancers, chromoplexy breakpoints and reciprocal translocations shared similar patterns.^20^ For example, they were both enriched in late-replicated regions. In sharp contrast, chromoplexy breakpoints in pediatric brain tumors were enriched in early-replicated regions while reciprocal translocations did not display any bias in replication timing (Figure 3; Figure S4). In addition, chromoplexy breakpoints in pediatric brain tumors were also enriched in GC-rich regions and near telomeres, while reciprocal translocations were enriched in AT-rich regions and not enriched toward either centromeres or telomeres (Figure 3; Figure S4). These results suggested that chromoplexy in pediatric brain tumors may form through a different mechanism than reciprocal translocations. Cycle of templated insertion breakpoints in pediatric brain tumors had little association with most genomic features except for proximity to telomeres (Figure 3; Figure S4).

Simple SV breakpoint distributions of pediatric brain tumors were also quite distinct from those of adult cancers. In adult cancers, deletions were enriched in early-replicated regions, and TDs and Unbal tras were enriched in late-replicated regions.^20^ In contrast, in pediatric brain tumors, small deletions (“Del0” and “Del1”) were not associated with replication timing, large deletions (“Del2”) were enriched in late-replicated regions, and TDs as well as Unbal tras were enriched in early-replicated regions (Figure 3; Figure S4). In adult cancers, all deletions, regardless of their size, were enriched in AT-rich regions;^20^ in pediatric brain tumors, small deletions (“Del1”) were enriched in GC-rich regions; and large deletions (“Del2”) were enriched in AT-rich regions (Figure 3; Figure S4). In adult cancers, TDs were significantly closer to Alu elements,^20^ and in pediatric brain tumors, TDs) were farther away from L1s, LTR transposons, and DNA transposons (Figure 3). In adult cancers, small and large deletions were depleted from topologically associated domain (TAD) boundaries.^20^ However, in pediatric brain tumors, only large deletions (“Del2”), but not small deletions (“Del0” and “Del1”), were depleted from TAD boundaries (Figure 3).

Taken together, our results suggest that the mechanisms of formation of complex and simple somatic SVs in pediatric brain tumors are likely to be different from those in adult cancers.

Next, we sought to identify mutations associated with genome instability. As expected, somatic *TP53* mutations in HGGs were associated with “Chr bridge,” “Large loss,” and cycle of templated insertions as well as six simple SV signatures: “Unbal inv,” “Unbal tra,” “Large mixed,” “TD,” “Del1,” and “Del2” (Figures S5A and S5B). A total of 17 patients carried germline deleterious or truncating variants in *TP53* (Table S4), and these variants were associated with almost all SV signatures when all tumor types were combined (Figure S5E). Somatic *ATRX* mutations in HGGs were associated with “Large mixed” signatures (Figure S5D). *TP53* and *ATRX* are known to play important roles in DNA damage repair. In addition, somatic *H3F3A* mutations in HGGs were associated with cycle of templated insertions (Figure S5C), suggesting that histones may play important roles in genome instability in pediatric brain tumors. Histone 3 is the fundamental unit of chromatin that organizes DNA; therefore, histone mutations are also likely to impact DNA damage repair. Because no external mutagenetic process has been reported in HGGs, it is likely that the mutations in *TP53*, *ATRX*, and *H3F3A* lead to various types of DNA damage repair deficiencies and are associated with multiple SV signatures. No other mutations were associated with any SV signatures in any other pediatric brain tumors. In contrast, no somatic mutations in any genes were associated with any SV signatures in adult GBM or LGG. Furthermore, in adult cancers, small deletions are associated with *BRCA2* mutations, and small and large TDs are associated with *BRCA1* and *CDK12* mutations.^12,20^ In the 741 non-hypermutated pediatric brain tumors, only one sample carried a *BRCA1* mutation, and another sample carried a *BRCA2* mutation. This again suggested that the mechanisms of formation of deletions and TDs in pediatric brain tumors are likely to be different from those of adult cancers.

### SV breakpoint sequences

**Figure 4.**
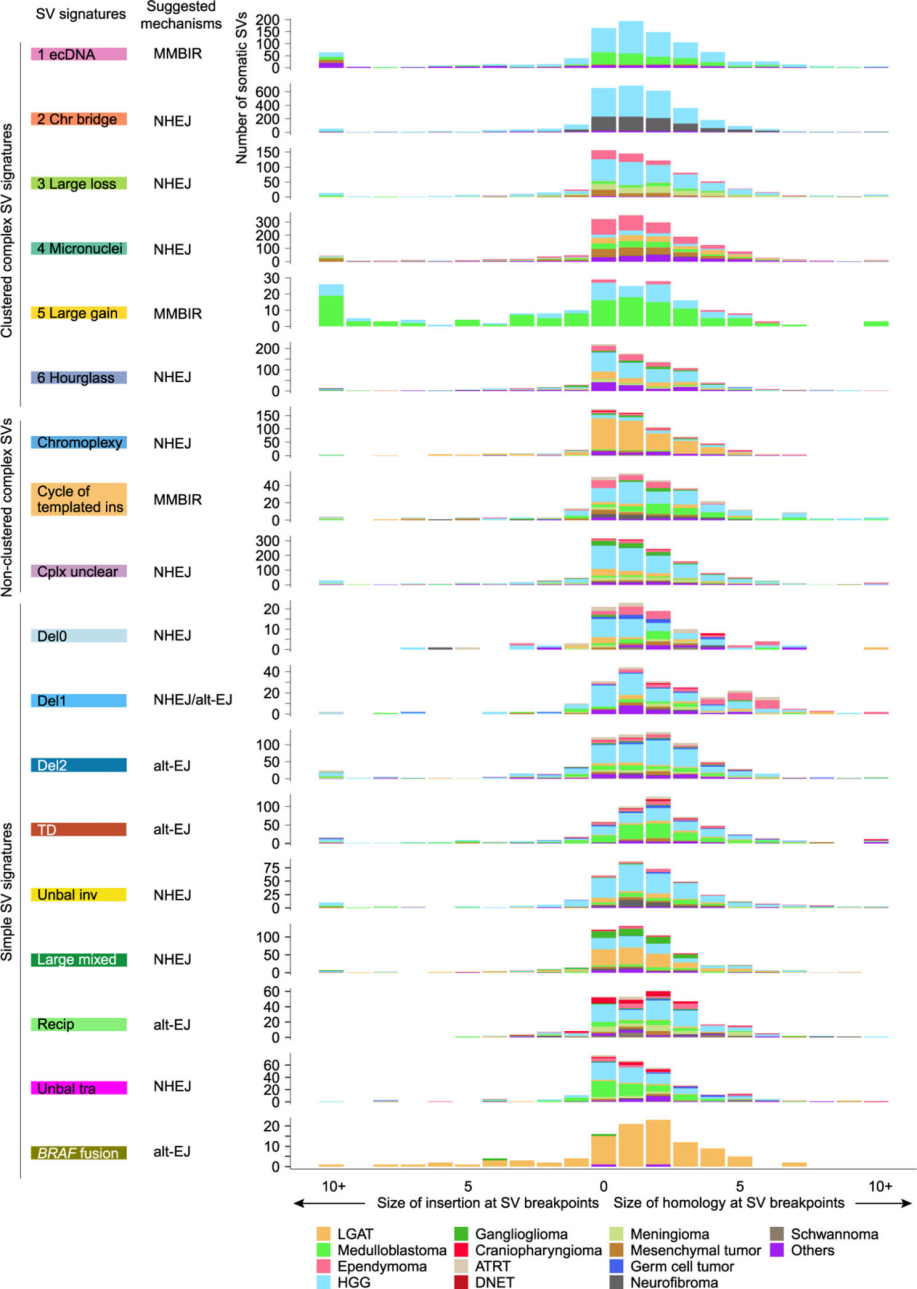
SV breakpoint homology The distributions (x axis) of homology and insertion at SV breakpoints are shown for all SV signatures (y axis). The putative DNA repair mechanisms are inferred from the sizes of homology and insertion and annotated next to the signatures. The bars indicate number of somatic SVs and are colored by tumor type. MMBIR, microhomology-mediated break-induced repair; NHEJ, non-homologous end joining; alt-EJ, alternative end joining.

We then investigated microhomology and insertion sequences at the SV breakpoints across SV signatures. SVs result from erroneous repair of DNA double-strand breaks or replication errors. Various repair pathways are involved,^41,42^ such as non-homologous end joining (NHEJ), alternative end joining (alt-EJ), and microhomology-mediated break-induced repair (MMBIR). NHEJ usually ligates blunt DNA ends or ends with short 1- to 4-bp homology. Alt-EJ often uses slightly longer homology for repair. MMBIR is considered a replication-based template-switching mechanism, and non-template insertions are frequently present at the breakpoints.^33^ Several SV signatures in pediatric brain tumors, including “Large loss,” “Hourglass,” chromoplexy, and “Unbal tra,” had a majority of SV breakpoints that were blunt DNA ends (no homology nor insertion at the breakpoints) (Figure 4). This suggested that these SVs are likely to form through NHEJ. Some other signatures, such as “Chr bridge,” “Micronuclei,” “Del0,” “Del1,” “Unbal inv,” and “Large mixed,” had slightly longer homology at the breakpoints, with 1-bp microhomology being the most frequent and also consistent with NHEJ (Figure 4). In addition, the “Del2,” “TD,” “Recip,” and “*BRAF* fusion” signatures had 2-bp microhomology being the most frequent (Figure 4), suggesting that alt-EJ might play more important roles in these SVs. The observation of extended microhomology in the “Recip” signature was consistent with breakpoints found in the Philadelphia chromosome, the most prevalent reciprocal translocation in leukemia, often involving 2- to 8-bp microhomology.^43^ Although previous studies have proposed that chromoplexy forms in a manner similar to reciprocal translocation,^31,32^ our observation of chromoplexy and reciprocal translocation breakpoints with different microhomology patterns again suggested that they may form via different mechanisms. Furthermore, a fraction of SVs in “Del1” had 5-bp or longer microhomology (Figure 4) especially in ependymomas, suggesting that alt-EJ is the dominant mechanism in ependymoma. Intriguingly, “ecDNA” and “Large gain” signatures had frequent inserted sequences that were more than 10 bp (Figure 4), suggesting possible involvement of replication-based mechanisms, such as MMBIR. We note that the putative repair mechanisms were nominated for SV signatures based on the overall pattern of homology and the most dominant homology size. Further study is necessary to better understand the mechanisms of DNA damage and damage repair leading to the SVs.

### SV hotspots and tumor drivers

**Figure 5.**
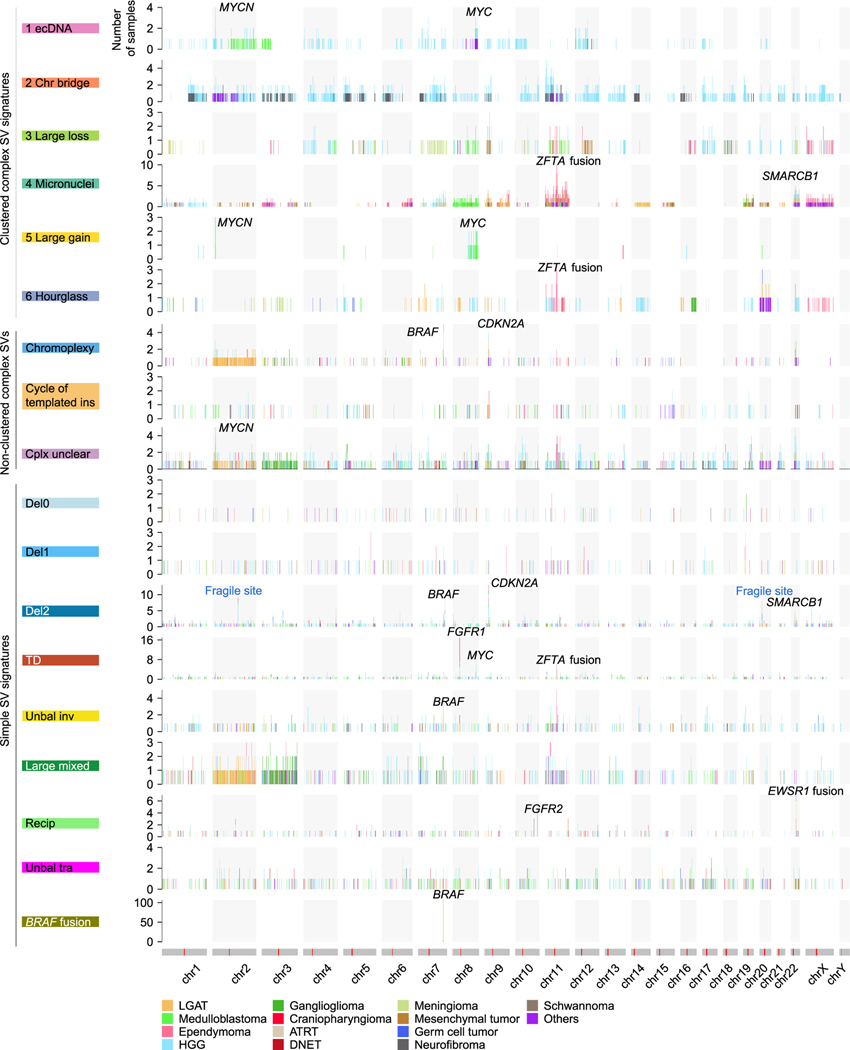
SV breakpoint hotspots SV breakpoint frequencies are shown for the entire reference genome (x axis) across different SV signatures (y axis). Chromosome models are shown as gray bars, with red lines indicating locations of centromeres at the bottom. Hotspots containing known oncogenes, tumor suppressors, and fragile sites are annotated.

Hotspots of SV breakpoints often represent genetic alterations under positive selection and genes driving diseases. After binning the reference genome into 1-Mb windows and counting SV occurrences, we found different SV signatures having quite distinct hotspots. *MYCN* and *MYC* are two frequently amplified oncogenes in pediatric brain tumors.^22^ *MYCN* was amplified exclusively by “ecDNA” in HGGs and mainly by “Large gain” in medulloblastomas (Figure 5). “ecDNA” and “Large gain” were the most abundant clustered complex SV signatures in HGGs and medulloblastomas, respectively. Both signatures converged on *MYCN* amplifications, which are the primary oncogenic events in HGGs and medulloblastomas. *MYCN* was also amplified by non-clustered complex SV with an unclear pattern (“Complex unclear”) in various tumor types (Figure 5). TDs can also amplify DNA. However, *MYCN* was not amplified by the “TD” signature in any samples, whereas *MYC* was amplified by the “TD” signature in a few HGGs and medulloblastomas (Figure 5). In addition, *FGFR1* was frequently amplified by the “TD” signature in DNETs (Figure 5). DNETs had very few somatic SVs and did not carry any clustered or non-clustered complex SVs. “TD” was the dominant SV signature in DNETs. These findings suggested that “TD” and *FGFR1* amplifications were the major oncogenic events in DNETs. Furthermore, chromoplexy and “Del2” frequently disrupted *CDKN2A* in various tumor types (Figure 5). The “*BRAF* fusion” signature produced *KIAA1549*-*BRAF* fusions mainly in LGATs (Figure 5). The “Recip” signature often led to *EWSR1* fusions in various tumor types (Figure 5).

**Figure 6.**
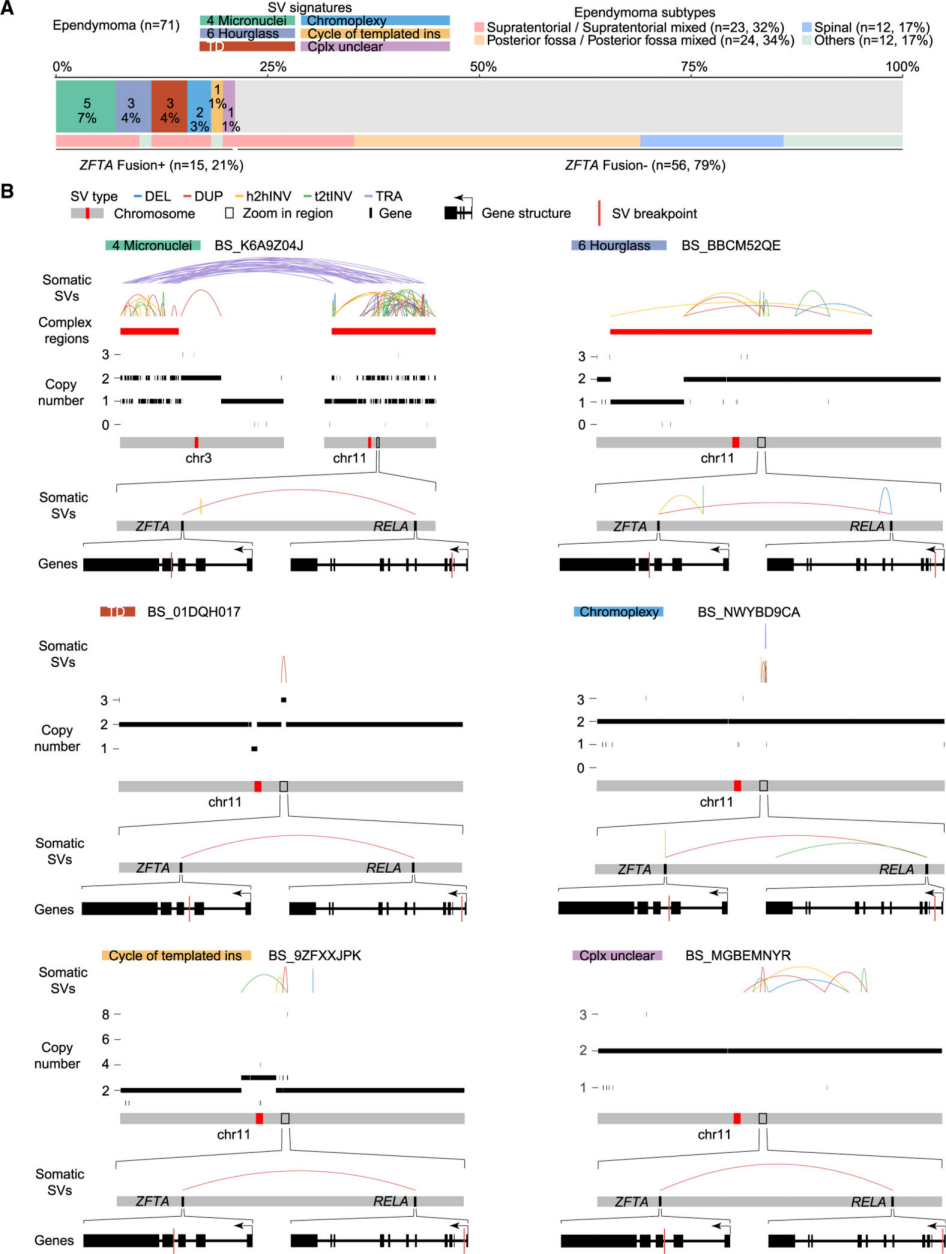
*ZFTA* fusions in ependymomas (A) The prevalence of *ZFTA* fusions in 71 ependymomas. Samples are colored by signatures of the SVs resulting in *ZFTA* fusions and ependymoma subtypes. (B) Six examples of *ZFTA* fusions resulting from different SV signatures. SV signatures and sample IDs are shown on the top. Somatic SVs, regions of complex SVs, and copy number profiles are displayed in the same scheme as Figure 1C. The *ZFTA* and *RELA* regions are magnified, and the *ZFTA* gene and *RELA* gene are further magnified, respectively. Gene structures are shown at the bottom of six examples. Within gene structures, the SV breakpoints that lead to *ZFTA*-*RELA* fusions are shown as red vertical lines. The directions of gene transcription are indicated by arrows. See also Table S5.

Interestingly, multiple SV signatures harbored hotspots on chromosome 11, and the hotspots were only found in ependymomas (Figure 5). *C11orf95*-*RELA* fusions are the major oncogenic events in 70% of supratentorial ependymomas.^44^ Recently, the WHO recommended the use of the *ZFTA* (*C110rf95*) fusion to classify supratentorial ependymoma instead of the *RELA* fusion because *ZFTA* can fuse to other partners as well.^28^ There were 71 ependymomas in our cohort, and 23 of them (32%) were supratentorial (Figure 6A; Table S5). Among them, 13 (57%) carried *ZFTA* fusions (Figure 6A). There were 2 other *ZFTA* fusion-positive ependymomas classified as “Others” (Figure 6A). Twelve of 15 *ZFTA* fusions were driven by complex SVs, and three were driven by TDs (Figure 6A). Among the 12 fusions resulting from complex events, 5 were micronucleus-induced chromothripsis (“Micronuclei”), 3 were hourglass chromothripsis (“Hourglass”), and 4 were non-clustered complex SVs (Figures 6A and 6B). Some of the complex SVs involved the entire chromosome 11 (BS_K6A9Z04J), whereas others only affected a small region in chromosome 11 (BS_NWYBD9CA) (Figure 6B). These results showed that there are diverse mechanisms generating genome instability in ependymomas that share the oncogenic consequence of forming *ZFTA* fusions. The most prevalent complex SVs in ependymomas were micronucleus-induced chromothripsis events caused by erroneous chromosomal segregation. It is possible that the frequent complex SVs in ependymomas are due to frequent chromosomal segregation errors, but the rareness of aneuploidy in ependymomas^45^ makes it unlikely that chromosomal segregation errors frequent events in ependymomas. The fact that TDs are sufficient to produce gene fusions, such as *ZFTA* fusions and *BRAF* fusions and the finding that most somatic SVs in ependymomas involving chromosome 11 were complex SVs suggest that other genes altered by SVs may contribute to ependymoma tumorigenesis as well. The “Unbal inv” signature also had a hotspot in a similar region on chromosome 11 (Figure 5). Three ependymomas had Unbal invs in the gene *MARK2*, which is next to *ZFTA*, and no fusions were formed. Whether this “Unbal inv” hotspot in ependymoma reflects oncogenic events remains unclear.

In a significant fraction of pediatric brain tumors, the cancer driver SVs were the sole SVs of the corresponding signatures. These signatures did not produce additional passenger SVs in those samples. For example, 88 LGATs were driven by *BRAF* fusions, and there was only one SV within the “*BRAF* fusion” signature in those samples (Figure 2B). Similarly, three ependymomas carried *ZFTA* fusions caused by the “TD” signature, and there was only one SV within the “TD” signature in two of these three samples (Figures 2B; Figure 5). In addition, nine DNETs had *FGFR1* amplifications resulting from the “TD” signature, and there was only one SV within the “TD” signature in all nine samples (Figures 2B; Figure 5). These results indicated that the molecular mechanisms leading to these disease-driving SVs are not highly active in tumor-initiating cells. Cells independently acquire SVs through these mechanisms at very low rates, and cells that have acquired the SVs that alter the major disease-driving genes eventually outcompete other cells to become tumors.

### SVs associated with clinical properties

**Figure 7.**
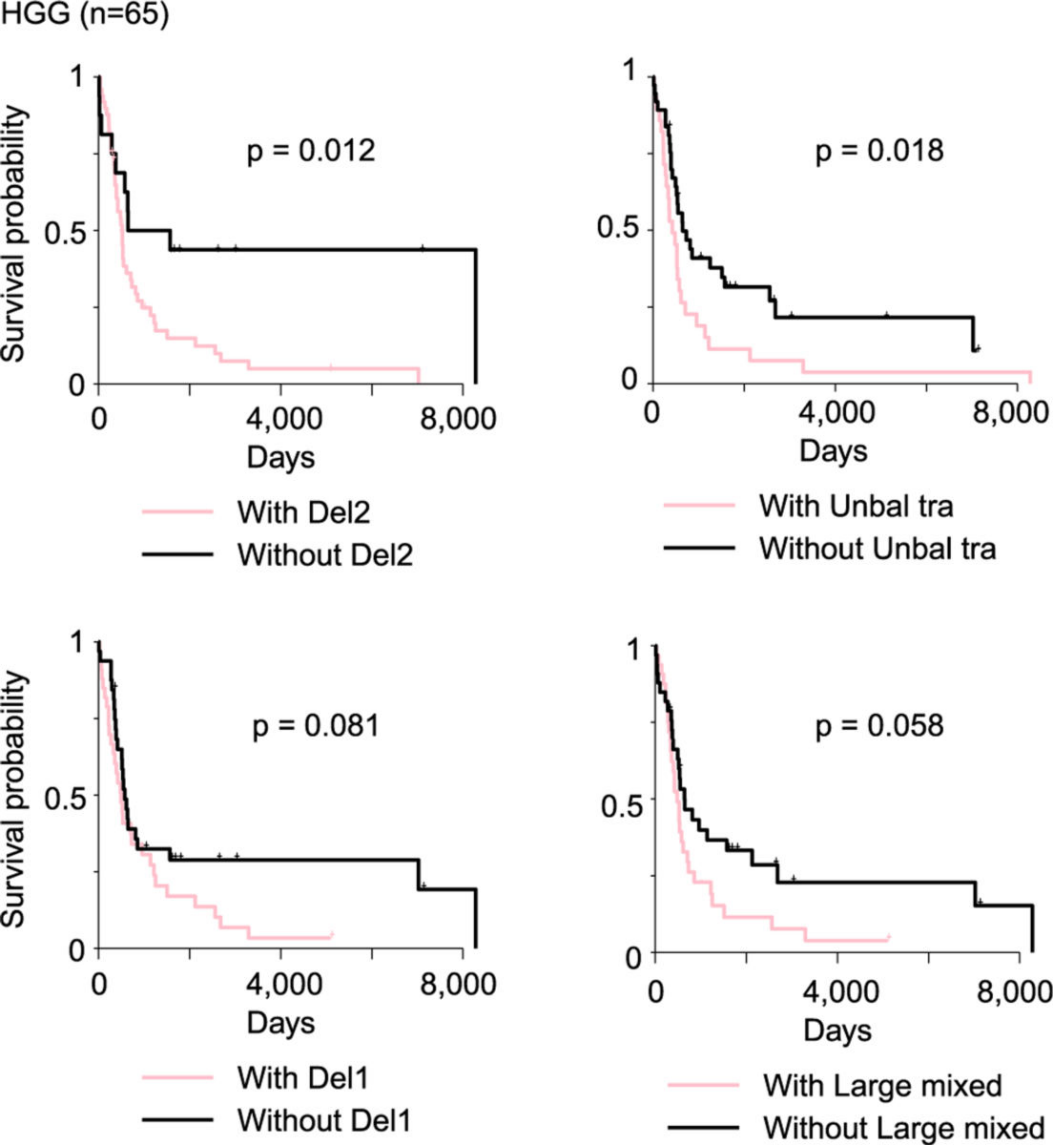
SV signatures associated with patient survival Kaplan-Meier survival curves for HGG patients, stratified by the presence or absence of four simple SV signatures (“Del2,” “Unbal tra,” “Del1,” and “Large mixed”) are shown. The p values are calculated by log rank test. See also Figures S6 and S7.

Next, we sought to evaluate whether somatic SVs affect patient survival in pediatric brain tumors. Chromothripsis has been associated with worse patient survival in several previous studies.^46–49^ However, we did not observe any complex SV signatures associated with patient survival in HGGs, LGATs, medulloblastomas, or ependymomas (Figure S6A). We reason that clustered complex SVs are one-time events and are likely rare during tumorigenesis. They often converge on a few major cancer-driving genes. The complex SVs being observed in tumor genomes are likely under positive selection. Therefore, the presence and absence of clustered complex SVs do not have significant impact on patient survival. HGG patients with “Del2” and “Unbal tra” signatures in their tumors had significantly worse survival, and those with “Del1” and “Large mixed” signatures had marginally worse survival (Figure 7). HGGs were the tumors with the highest abundance of simple SVs, which suggested that the SV-forming mechanisms are relatively more active in tumor-initiating cells of HGGs than those of other tumor types. No simple SV signatures were associated with patient survival in other tumor types. It is possible that simple SV-forming mechanisms are not very active in tumor types other than HGGs. In addition, it has also been reported that somatic SVs in HGGs activate oncogenes such as *EGFR*, *MET*, and *PDGFRA*.^22^ Therefore, patients carrying simple SVs in their tumors have worse survival in HGGs (Figure 7). In adult brain cancers, there was no difference in survival between cancers with and without clustered complex SVs in GBM (Figure S6B). Although LGG patients with “Large loss” and “Micronuclei” signatures in their tumors had worse survival, the difference was only driven by two patients (Figure S6B). Interestingly, in adult GBM, patients with “TD3,” “Unbal tra,” and “Chromoplexy” signatures in their tumors had significantly better survival, whereas in adult LGG, patients with “Del3,” “Fragile site,” and “Unbal tra” signatures in their tumors had significantly worse survival (Figure S6C).

We then assessed whether somatic SVs were associated with age of diagnosis. Because age of diagnosis is highly correlated with tumor types, we separately considered three tumor types for which the sample sizes were sufficient for statistical tests. In ATRT, “Micronuclei” and cycle of templated insertion were associated with older patients (Figure S7A). Because the “Micronuclei” signature had a hotspot at the *SMARCB1* locus in ATRT (Figure 5), and loss of *SMARCB1* is known to promote ATRT,^50^ it is possible that disruption of *SMARCB1* by “Micronuclei” is a rate-limiting step, and, therefore, patients were diagnosed at older ages. Genes altered by cycle of templated insertion in ATRT were unclear. In HGGs, complex SVs were not associated with age of diagnosis, whereas various simple SV signatures, including “Del1,” “Del2,” “TD,” “Unbal inv,” and “Large mixed,” were associated with diagnosis in older patients (Figure S7B). Because these SV signatures are likely to be driven by DNA damage repair deficiencies, and mutations in *TP53*, *ATRX*, and *H3F3A* are required to induce the repair deficiencies, disease progression is likely to take longer in these patients. In LGAT, “*BRAF* fusion” was associated with younger patients (Figure S7C), which was consistent with *BRAF* fusion being a primary cancer driver.

### Limitations of the study

Pediatric brain tumor classification is challenging because of heterogeneity of the diseases. The 2021 WHO classification of CNS tumors has a hierarchical structure.^28^ We chose tumor type classification primarily by sample size. Some tumor types we used in Figure 1A can be further classified into more detailed types. For example, embryonal tumors can be classified into medulloblastoma and “other CNS embryonal tumors.” “Other CNS embryonal tumors” can be further classified into ATRT, embryonal tumors with multilayered rosettes, etc. Although medulloblastoma and ATRT are not at the same level of classification, we still used these two types because there were sufficient samples that allowed us to compare them with other types. For somatic SV calling, because we did not have access to the patient samples, we were unable to directly assess the quality of variants using experimental approaches, such as PCR and Sanger sequencing. Therefore, we conducted a comparative analysis using CNV breakpoints to infer the quality of SVs. In addition, our study reported numerous associations between somatic SVs in pediatric brain tumors and genomic and clinical properties. However, statistical association does not imply causal relationship. Caution should be taken when interpreting the associations.

## STAR★METHODS

### RESOURCE AVAILABILITY

#### Lead contact

Further information for resources will be fulfilled by the Lead Contact, Lixing Yang (lixingyang@uchicago.edu).

#### Materials availability

This study did not generate new unique reagents.

#### Data and code availability

The raw normal and tumor whole-genome sequencing data and germline SNVs for 744 pediatric brain tumor patients can be downloaded from CAVATICA (https://cavatica.sbgenomics.com/). Sample characteristics, clinical data, somatic SNV, and somatic CNV data can be retrieved from OpenPBTA (https://github.com/AlexsLemonade/OpenPBTA-analysis). The consensus somatic SVs for PCAWG samples along with clinical information including diagnosis and survival data of adult brain tumors can be obtained from the PCAWG consortium (https://www.sevenbridges.com/case-studies/pcawg/).

This paper does not report original code.

Any additional information required to reanalyze the data reported in this work paper is available from the lead contact upon request.

### METHOD DETAILS

#### Sample and data collection

The raw normal and tumor whole-genome sequencing data and germline SNVs for 744 pediatric brain tumor patients were downloaded from CAVATICA (https://cavatica.sbgenomics.com/). Sample characteristics, clinical data, somatic SNV, and somatic CNV data were retrieved from OpenPBTA^51^ (https://github.com/AlexsLemonade/OpenPBTA-analysis). The consensus SV data, as called by four algorithms, along with clinical information including diagnosis and survival data of adult brain tumors, were obtained from the PCAWG consortium.

Gene annotation was obtained from ENSEMBL (GRCh38.p13) (https://useast.ensembl.org/index.html). Non-B DNA structures including A-phased repeats, direct repeats, G-quadruplex forming repeats, inverted repeats, mirror repeats, short tandem repeats, and Z-DNA motifs were downloaded from non-B DB^52^ (https://nonb-abcc.ncifcrf.gov/apps/nBMST/default/); Alu, L1, L2, LTR, MIR, simple repeat, transposon, and low complexity repetitive elements, as well as the coordinates of centromeres, telomeres, and CpG islands, were obtained from UCSC (https://hgdownload.soe.ucsc.edu/goldenPath/hg38/database/); consensus estimates of the topologically associated domains (TADs) were downloaded from TAD Map^53^ (https://cb.csail.mit.edu/cb/tadmap/); ChIP-seq data of epigenetic markers H3K4me1, H3K9me3, H3K36me3, H3K4me3, H3K27me3, H3K9ac, H3K4me2, H3K79me2, H3K27ac, and H4K20me1 from human astrocytes were downloaded from ENCODE^54^ (https://www.encodeproject.org/). The Wavelet-smoothed signal of replication timing data for the cell lines BG02ES and SK-N-SH were downloaded from UCSC (https://genome.ucsc.edu/cgi-bin/hgFileUi?db=hg19&g=wgEncodeUwRepliSeq). The fragile site regions were obtained from a previous study,^20^ and the co-ordinates were lifted over to hg38. All coordinates in this study were based on the hg38 genome assembly unless otherwise noted.

#### Tumor classifications

The histological classifications of pediatric brain tumor samples were determined based on diagnosis, pathological examination, and histological examination according to the 2021 WHO classifications of pediatric brain tumors.^28^ For tumor types with at least 10 samples, we attempted to subclassify them. Gliomas were subclassified into high-grade gliomas (HGGs), low-grade astrocytic tumors (LGATs), ependymomas, dysembryoplastic neuroepithelial tumors (DNETs), and gangliogliomas. Embryonal tumors were subclassified into medulloblastomas, and atypical teratoid rhabdoid tumors (ATRTs). Cranial and paraspinal nerve tumors included schwannomas and neurofibromas. Germ cell tumors included teratomas, germinomas, and other germ cell tumors. Mesenchymal non-meningothelial tumors included hemangioblastomas, Ewing sarcomas, rhabdomyosarcomas, myofibroblastomas, and other sarcomas. Meningiomas, craniopharyngiomas, and choroid plexus tumors were independently classified. Tumor types with less than 10 samples were all classified into “Others”. Ependymomas were further stratified into distinct subtypes based on the primary sites.

#### Somatic SV calling and filtering

Manta (https://github.com/Illumina/manta), Meerkat (https://github.com/guru-yang/Meerkat), and Delly (https://github.com/dellytools/delly) were used for somatic SV detection. SVs called by Manta were obtained from OpenPBTA. Meerkat was run as suggested.^26^ Delly was run with default settings. For SVs detected by Delly, at least four supporting read pairs and split read combined were required for SVs less than 500 bp. For all three SV detection algorithms, only SVs located in canonical chromosomes (chr1–22, X, Y) were retained. SVs identified by different algorithms were considered identical if their two breakpoints were on the same chromosomes, with the same orientations and within 10 bp. SVs identified by two or more algorithms were considered high-confidence SVs and used in the subsequent analysis. Deletions with both breakpoints within 3 bp of exon-intron boundaries of the same genes were excluded from further analysis.

Somatic CNVs were used to assess the quality of somatic SVs. For each SV, if the distances of both SV breakpoints were less than 1 kb to the nearest CNV breakpoints, the SV was considered validated.

#### Complex SVs and their signatures

We used Starfish^21^ (https://github.com/yanglab-computationalgenomics/Starfish) to detect clustered complex SVs and classified them into six signatures. In cases where reported gender and germline estimated sex were inconsistent, gender identity was recorded as unknown for signature detection. After removing clustered complex SVs, we used ClusterSV^20^ (https://github.com/cancerit/ClusterSV) to identify non-clustered complex SVs. Non-clustered complex SVs include chromoplexy, cycle of templated insertions, and complex unclear.

#### Simple SV signatures

After removing clustered and non-clustered complex SVs, the remainder were simple SVs including four major categories: deletions, tandem duplications, inversions, and translocations. Deletions and tandem duplications with breakpoints falling within fragile site regions were classified as fragile site deletions and fragile site tandem duplications, respectively. The remaining deletions and tandem duplications were classified into 18 subcategories based on their sizes. Inversions and translocations were further subclassified into reciprocal inversions, fold-back inversions, unbalanced inversions, reciprocal translocations, and unbalanced translocations. Unbalanced inversions and reciprocal inversions were classified into 3 and 5 subcategories based on their sizes, respectively. Fold-back inversions, unbalanced translocations, and reciprocal translocations were three independent subcategories. As a result, all simple SVs were classified into 49 subcategories and SigProfilerExtractor^34^ (https://github.com/AlexandrovLab/SigProfilerExtractor) with default parameters was used to extract simple SV signatures. According to the final signatures we chose, deletions smaller than 1 kb were assigned as “Del0”; deletions ranging in size from 1 kb to 5 kb were classified as “Del1”; fragile site deletions, fragile site tandem duplications, and deletions sized in 5 kb–10 Mb were assigned as “Del2”; tandem duplications between 1 Mb and 2.5 Mb with breakpoints located within the *BRAF* region were classified as *BRAF* fusion signature; other <10 Mb tandem duplications were classified as “TD”; foldback inversions and unbalanced inversions sized between 50 kb and 5 Mb were categorized as “Unbal inv”; deletions and tandem duplications larger than 10 Mb, as well as reciprocal unbalanced inversions larger than 5 Mb, were classified as “Large mixed”; reciprocal inversions and reciprocal translocations were categorized as “Recip”; and unbalanced translocations were classified as “Unbal tra”. Another algorithm, signeR was also used to extract simple SV signatures using default settings.^35^

#### Genomic feature tests

For each observed somatic SV, we generated four random SVs of the same size and type on the same chromosome as observed SVs. All observed and randomized breakpoints were annotated with genomic features. Bedtools was used to compute the GC content within a ±50 bp interval of each SV breakpoint. The distances in kilobases (kb) from the breakpoints to the nearest Non-B DNA structures, repetitive elements, and CpG islands were logarithmically transformed, with the distances set to 0 if breakpoints were within any of the aforementioned elements. The distances in megabases (Mb) from the breakpoints to centromeres and telomeres and the distances (kb) to the closest TAD boundaries were also transformed to log scale. The SV breakpoints were annotated by signal -log10(p values) for different epigenetic modifications. The replication timing data for cell lines BG02ES and SK-N-SH were quantile normalized. The SV breakpoints were lifted over to hg19 since the replication timing data were based on hg19. The replication timing values were then annotated for each SV breakpoint. Breakpoints of observed SVs and randomized SVs were tested as described in the previous study.^20^ Briefly, scores of SV breakpoints for all genomic features were rescaled from 0 to 1. The distributions of scores between observed breakpoints and randomized breakpoints were compared using two-sided Kolmogorov-Smirnov test. False discovery rates (FDRs) were computed using the Benjamini-Hochberg procedure and 0.1 FDR cutoff was used to determine significant associations. Homology and insertion size at the SV breakpoints were provided by Meerkat and Manta.

#### Hotspot analysis

The reference genome was divided into 1 Mb non-overlapping bins. The number of samples with SV breakpoints in each bin was counted for each SV signature. A sample with multiple SV breakpoints of the same SV signatures falling within the same bin was only counted once.

#### Mutation test

Only protein-altering somatic SNVs and indels were considered in the test of the associations between SV signatures and somatic mutations, including missense mutations, splice site mutations, frameshift indels, nonsense mutations, translation start site mutations, and nonstop mutations. Three HGG samples (BS_20TBZG09, BS_02YBZSBY, and BS_VW4XN9Y7) with hypermutation were excluded. The tests were performed within tumor types. Protein-coding genes with mutation frequencies ≥ 5% in each tumor type were analyzed. Samples were classified into two categories based on the presence and absence of the SV signatures. Fisher’s exact test was used to calculate p values. FDRs were computed using the Benjamini-Hochberg procedure. FDR <0.1 was considered as significant.

Samples with germline deleterious missense SNVs predicted by SIFT,^55^ splice region SNVs and frameshift indels in *TP53* were categorized as “deleterious”, while samples with other *TP53* SNVs and indels were categorized as “benign”. The remaining samples were classified as “wild-type”. The test between germline *TP53* variants and SV signatures was conducted across all 744 PBTA brain tumors. Fisher’s exact test was used to calculate p values. FDRs were computed using the Benjamini-Hochberg procedure. FDR <0.1 was considered as significant.

#### Survival analysis

Since patient survival differs dramatically across tumor types, survival analysis was only performed within, but not across tumor types. For clustered complex SV signatures, samples with only one signature were assigned to the corresponding signatures; samples with more than one clustered complex SV signatures were classified into “Mixed”; and samples without any clustered complex SV were assigned into “None”. For simple SV signatures, samples were classified based on the presence and absence of the signatures. Log rank test was used to calculate p values.

#### Age of diagnosis analysis

Age of diagnosis analysis was exclusively conducted within specific tumor types. Regarding clustered complex SV signatures, samples with only one signature were allocated to their respective signatures; samples harboring multiple clustered complex SV signatures were categorized as “Mixed”; and samples lacking any clustered complex SVs were classified as “None”. As for simple SV signatures, samples were categorized based on the presence or absence of the signatures. ANOVA test was used to assess differences among multiple groups, and Student’s t test was used for comparisons between two groups. The FDRs were adjusted using the Benjamini-Yekutieli method.

#### QUANTIFICATION AND STATISTICAL ANALYSIS

Data were analyzed with R 4.1.1. For the genomic feature tests, the distributions of genomic feature scores between observed breakpoints and randomized breakpoints were compared using two-sided Kolmogorov-Smirnov test. FDRs were computed using the Benjamini-Hochberg procedure. Statistical significances were represented by the size of dots in Figure 3. For the mutation tests, Fisher’s exact test was used to calculate p values. FDRs were computed using the Benjamini-Hochberg procedure and 0.1 FDR cut-off was used to determine significant associations. For survival analysis, Log rank test was used to calculate p values, p < 0.05 was considered as significant. For age of diagnosis, ANOVA test was used to assess differences among complex SV signature groups, and Student’s t test was used to compare two groups. FDRs were adjusted using the Benjamini-Yekutieli method and 0.1 FDR cutoff was used to determine significant associations.

## Supplementary Material

1

2

3

4

## Figures and Tables

**Table:** KEY RESOURCES TABLE

REAGENT or RESOURCE	SOURCE	IDENTIFIER

Deposited data		

Pediatric brain tumor raw sequencing data, Manta called structural variations, germline SNV	CAVATICA	https://cavatica.sbgenomics.com/
Sample characteristics, clinical data, manta-called structural variations, somatic SNVs, CNVs	Shapiro et al.^51^	https://github.com/AlexsLemonade/OpenPBTA-analysis
Consensus SV data along with clinical information including diagnosis and survival data of adult brain tumors	PCAWG consortium^25^	https://www.sevenbridges.com/case-studies/pcawg/
Human reference genome (GRCh38.p13)	ENSEMBL	https://useast.ensembl.org/index.html
Non-B DNA structures	Cer et al.^52^	https://nonb-abcc.ncifcrf.gov/apps/nBMST/default/
Alu, L1, L2, LTR, MIR, simple repeat, transposon, and low complexity repetitive elements, centromeres, telomeres, and CpG islands	UCSC	https://hgdownload.soe.ucsc.edu/goldenPath/hg38/database/
Topologically associated domains	Singh et al.^53^	https://cb.csail.mit.edu/cb/tadmap/
ChIP-seq data of epigenetic markers from human astrocytes	Zhang et al.^54^	https://www.encodeproject.org/
Replication timing data for the cell lines BG02ES and SK-N-SH	UCSC	https://genome.ucsc.edu/cgi-bin/hgFileUi?db=hg19&g=wgEncodeUwRepliSeq
Fragile site regions	Li et al.^20^	Table S5; https://www.nature.com/articles/s41586-019-1913-9

Software and algorithms		

Meerkat 0.189	Yang et al.^26^	https://github.com/guru-yang/Meerkat
Delly 1.1.6	Rausch et al.^27^	https://github.com/dellytools/delly
Starfish	Bao et al.^21^	https://github.com/yanglab-computationalgenomics/Starfish
ClusterSV	Li et al.^20^	https://github.com/cancerit/ClusterSV
SigProfilerExtractor 1.2.0	Islam et al.^34^	https://github.com/AlexandrovLab/SigProfilerExtractor
R 4.1.1	R-Project for Stat computing	https://www.npackd.org/p/r/4.1.1
bedtools 2.29.0	Bedtools	https://github.com/arq5x/bedtools2/releases
Samtools 1.10	Samtools	https://sourceforge.net/projects/samtools/files/samtools/1.10/

## Highlights

- High-confidence somatic SVs are detected from pediatric brain tumors
- Complex and simple SV signatures are decomposed
- Pediatric brain tumors exhibit distinct SV signatures and differ from adult cancers
- Multiple signatures often converge to alter major cancer driver genes

## References

[1] Ostrom QT, de Blank PM, Kruchko C, Petersen CM, Liao P, Finlay JL, Stearns DS, Wolff JE, Wolinsky Y, Letterio JJ, and Barnholtz-Sloan JS (2015). Alex’s Lemonade Stand Foundation Infant and Childhood Primary Brain and Central Nervous System Tumors Diagnosed in the United States in 2007–2011. Neuro Oncol. 16, x1–x36. 10.1093/neuonc/nou327. 25542864 PMC4277295

[2] Louis DN, Ohgaki H, Wiestler OD, Cavenee WK, Burger PC, Jouvet A, Scheithauer BW, and Kleihues P (2007). The 2007 WHO classification of tumours of the central nervous system. Acta Neuropathol. 114, 97–109. 10.1007/s00401-007-0243-4. 17618441 PMC1929165

[3] Gröbner SN, Worst BC, Weischenfeldt J, Buchhalter I, Kleinheinz K, Rudneva VA, Johann PD, Balasubramanian GP, Segura-Wang M, Brabetz S, (2018). The landscape of genomic alterations across childhood cancers. Nature 555, 321–327. 10.1038/nature25480. 29489754

[4] Stephens PJ, Greenman CD, Fu B, Yang F, Bignell GR, Mudie LJ, Pleasance ED, Lau KW, Beare D, Stebbings LA, (2011). Massive genomic rearrangement acquired in a single catastrophic event during cancer development. Cell 144, 27–40. 10.1016/j.cell.2010.11.055. 21215367 PMC3065307

[5] Zhang C-Z, Spektor A, Cornils H, Francis JM, Jackson EK, Liu S, Meyerson M, and Pellman D (2015). Chromothripsis from DNA damage in micronuclei. Nature 522, 179–184. 10.1038/nature14493. 26017310 PMC4742237

[6] Maciejowski J, Li Y, Bosco N, Campbell PJ, and de Lange T (2015). Chromothripsis and Kataegis Induced by Telomere Crisis. Cell 163, 1641–1654. 10.1016/j.cell.2015.11.054. 26687355 PMC4687025

[7] Alt FW, Zhang Y, Meng FL, Guo C, and Schwer B (2013). Mechanisms of programmed DNA lesions and genomic instability in the immune system. Cell 152, 417–429. 10.1016/j.cell.2013.01.007. 23374339 PMC4382911

[8] Hakim O, Resch W, Yamane A, Klein I, Kieffer-Kwon KR, Jankovic M, Oliveira T, Bothmer A, Voss TC, Ansarah-Sobrinho C, (2012). DNA damage defines sites of recurrent chromosomal translocations in B lymphocytes. Nature 484, 69–74. 10.1038/nature10909. 22314321 PMC3459314

[9] Bakhshi A, Jensen JP, Goldman P, Wright JJ, McBride OW, Epstein AL, and Korsmeyer SJ (1985). Cloning the chromosomal breakpoint of t(14;18) human lymphomas: clustering around Jh on chromosome 14 and near a transcriptional unit on 18. Cell 41, 899–906. 10.1016/S0092-8674(85)80070-2. 3924412

[10] Gostissa M, Yan CT, Bianco JM, Cogné M, Pinaud E, and Alt FW (2009). Long-range oncogenic activation of Igh-c-myc translocations by the Igh 3′ regulatory region. Nature 462, 803–807. 10.1038/nature08633. 20010689 PMC2802177

[11] Lee J-K, Choi Y-L, Kwon M, and Park PJ (2016). Mechanisms and Consequences of Cancer Genome Instability: Lessons from Genome Sequencing Studies. Annu. Rev. Pathol 11, 283–312. 10.1146/annurev-pathol-012615-044446. 26907526

[12] Nik-Zainal S, Davies H, Staaf J, Ramakrishna M, Glodzik D, Zou X, Martincorena I, Alexandrov LB, Martin S, Wedge DC, (2016). Landscape of somatic mutations in 560 breast cancer whole-genome sequences. Nature 534, 47–54. 10.1038/nature17676. 27135926 PMC4910866

[13] Fong PC, Boss DS, Yap TA, Tutt A, Wu P, Mergui-Roelvink M, Mortimer P, Swaisland H, Lau A, O’Connor MJ, (2009). Inhibition of Poly(ADP-Ribose) Polymerase in Tumors from BRCA Mutation Carriers. N. Engl. J. Med 361, 123–134. 10.1056/NEJMoa0900212. 19553641

[14] Farmer H, McCabe N, Lord CJ, Tutt ANJ, Johnson DA, Richardson TB, Santarosa M, Dillon KJ, Hickson I, Knights C, (2005). Targeting the DNA repair defect in BRCA mutant cells as a therapeutic strategy. Nature 434, 917–921. 10.1038/nature03445. 15829967

[15] Alexandrov LB, Nik-Zainal S, Wedge DC, Aparicio SAJR, Behjati S, Biankin AV, Bignell GR, Bolli N, Borg A, Børresen-Dale AL, (2013). Signatures of mutational processes in human cancer. Nature 500, 415–421. 10.1038/nature12477. 23945592 PMC3776390

[16] Alexandrov LB, Kim J, Haradhvala NJ, Huang MN, Tian Ng AW, Wu Y, Boot A, Covington KR, Gordenin DA, Bergstrom EN, (2020). The repertoire of mutational signatures in human cancer. Nature 578, 94–101. 10.1038/s41586-020-1943-3. 32025018 PMC7054213

[17] Degasperi A, Zou X, Amarante TD, Martinez-Martinez A, Koh GCC, Dias JML, Heskin L, Chmelova L, Rinaldi G, Wang VYW, (2022). Substitution mutational signatures in whole-genome–sequenced cancers in the UK population. Science 376, /SUPPL_FILE/SCIENCE.ABL9283_TABLES_S1_TO_S33.V1.ZIP. 10.1126/SCIENCE.ABL9283. PMC7613262 35949260

[18] Steele CD, Abbasi A, Islam SMA, Bowes AL, Khandekar A, Haase K, Hames-Fathi S, Ajayi D, Verfaillie A, Dhami P, (2022). Signatures of copy number alterations in human cancer. Nature 606, 984–991. 10.1038/s41586-022-04738-6. 35705804 PMC9242861

[19] Drews RM, Hernando B, Tarabichi M, Haase K, Lesluyes T, Smith PS, Morrill Gavarró L, Couturier DL, Liu L, Schneider M, (2022). A pan-cancer compendium of chromosomal instability. Nature 606, 976–983. 10.1038/s41586-022-04789-9. 35705807 PMC7613102

[20] Li Y, Roberts ND, Wala JA, Shapira O, Schumacher SE, Kumar K, Khurana E, Waszak S, Korbel JO, Haber JE, (2020). Patterns of somatic structural variation in human cancer genomes. Nature 578, 112–121. 10.1038/s41586-019-1913-9. 32025012 PMC7025897

[21] Bao L, Zhong X, Yang Y, and Yang L (2022). Starfish infers signatures of complex genomic rearrangements across human cancers. Nat. Can (Ott.) 3, 1247–1259. 10.1038/s43018-022-00404-y. PMC11077613 35835961

[22] Dubois FPB, Shapira O, Greenwald NF, Zack T, Wala J, Tsai JW, Crane A, Baguette A, Hadjadj D, Harutyunyan AS, (2022). Structural variants shape driver combinations and outcomes in pediatric high-grade glioma. Nat. Can. (Ott.) 2022 3, 994–1011. 10.1038/s43018-022-00403-z. PMC10365847 35788723

[23] Chen X, Schulz-Trieglaff O, Shaw R, Barnes B, Schlesinger F, Källberg M, Cox AJ, Kruglyak S, and Saunders CT (2016). Manta: rapid detection of structural variants and indels for germline and cancer sequencing applications. Bioinformatics 32, 1220–1222. 10.1093/bioinformatics/btv710. 26647377

[24] Zhang Y, Chen F, Donehower LA, Scheurer ME, and Creighton CJ (2021). A pediatric brain tumor atlas of genes deregulated by somatic genomic rearrangement. Nat. Commun 12, 937. 10.1038/s41467-021-21081-y. 33568653 PMC7876141

[25] ICGC/TCGA Pan-Cancer Analysis of Whole Genomes Consortium; Getz G, Korbel JO, Stuart JM, Jennings JL, Stein LD, Perry MD, Nahal-Bose HK, Ouellette BFF, Li CH, (2020). Pan-cancer analysis of whole genomes. Nature 578, 82–93. 10.1038/s41586-020-1969-6. 32025007 PMC7025898

[26] Yang L, Luquette LJ, Gehlenborg N, Xi R, Haseley PS, Hsieh C-H, Zhang C, Ren X, Protopopov A, Chin L, (2013). Diverse mechanisms of somatic structural variations in human cancer genomes. Cell 153, 919–929. 23663786 10.1016/j.cell.2013.04.010 PMC3704973

[27] Rausch T, Zichner T, Schlattl A, Stütz AM, Benes V, and Korbel JO (2012). DELLY: structural variant discovery by integrated pairedend and split-read analysis. Bioinformatics 28, i333–i339. 22962449 10.1093/bioinformatics/bts378 PMC3436805

[28] Louis DN, Perry A, Wesseling P, Brat DJ, Cree IA, Figarella-Branger D, Hawkins C, Ng HK, Pfister SM, Reifenberger G, (2021). The 2021 WHO Classification of Tumors of the Central Nervous System: a summary. Neuro Oncol. 23, 1231–1251. 10.1093/NEUONC/NOAB106. 34185076 PMC8328013

[29] Broniscer A, Baker SJ, West AN, Fraser MM, Proko E, Kocak M, Dalton J, Zambetti GP, Ellison DW, Kun LE, (2007). Clinical and molecular characteristics of malignant transformation of low-grade glioma in children. J. Clin. Oncol 25, 682–689. 17308273 10.1200/JCO.2006.06.8213

[30] Shoshani O, Brunner SF, Yaeger R, Ly P, Nechemia-Arbely Y, Kim DH, Fang R, Castillon GA, Yu M, Li JSZ, (2021). Chromothripsis drives the evolution of gene amplification in cancer. Nature 591, 137–141. 10.1038/s41586-020-03064-z. 33361815 PMC7933129

[31] Baca SC, Prandi D, Lawrence MS, Mosquera JM, Romanel A, Drier Y, Park K, Kitabayashi N, MacDonald TY, Ghandi M, (2013). Punctuated evolution of prostate cancer genomes. Cell 153, 666–677. 10.1016/j.cell.2013.03.021. 23622249 PMC3690918

[32] Berger MF, Lawrence MS, Demichelis F, Drier Y, Cibulskis K, Sivachenko AY, Sboner A, Esgueva R, Pflueger D, Sougnez C, (2011). The genomic complexity of primary human prostate cancer. Nature 470, 214–220. 21307934 10.1038/nature09744 PMC3075885

[33] Zhang F, Khajavi M, Connolly AM, Towne CF, Batish SD, and Lupski JR (2009). The DNA replication FoSTeS/MMBIR mechanism can generate genomic, genic and exonic complex rearrangements in humans. Nat. Genet 41, 849–853. 10.1038/ng.399. 19543269 PMC4461229

[34] Islam SA, Díaz-Gay M, Wu Y, Barnes M, Vangara R, Bergstrom EN, He Y, Vella M, Wang J, Teague JW, (2022). Uncovering novel mutational signatures by de novo extraction with SigProfilerExtractor. Cell Genomics 2, 100179. 10.1016/J.XGEN.2022.100179. 36388765 PMC9646490

[35] Rosales RA, Drummond RD, Valieris R, Dias-Neto E, and da Silva IT (2017). signeR: an empirical Bayesian approach to mutational signature discovery. Bioinformatics 33, 8–16. 10.1093/bioinformatics/btw572. 27591080

[36] Pfister S, Janzarik WG, Remke M, Ernst A, Werft W, Becker N, Toedt G, Wittmann A, Kratz C, Olbrich H, (2008). BRAF gene duplication constitutes a mechanism of MAPK pathway activation in low-grade astrocytomas. J. Clin. Invest 118, 1739–1749. 10.1172/JCI33656. 18398503 PMC2289793

[37] Jones DTW, Gronych J, Lichter P, Witt O, and Pfister SM (2011). MAPK pathway activation in pilocytic astrocytoma. Cell. Mol. Life Sci. 69, 1799–1811. 10.1007/S00018-011-0898-9. 22159586 PMC3350769

[38] Jones DTW, Hutter B, Jäger N, Korshunov A, Kool M, Warnatz HJ, Zichner T, Lambert SR, Ryzhova M, Quang DAK, (2013). Recurrent somatic alterations of FGFR1 and NTRK2 in pilocytic astrocytoma. Nat. Genet 45, 927–932. 10.1038/ng.2682. 23817572 PMC3951336

[39] Yang Y, Badura ML, O’Leary P, Robinson TM, Egusa EA, Zhong X, Swinderman JT, Li H, Zhang M, Kim M, (2023). Large tandem duplications in cancer result from transcription and DNA replication collision. Preprint at medRxiv. 10.1101/2023.05.17.23290140. PMC11671220 39558146

[40] Akdemir KC, Le VT, Chandran S, Li Y, Verhaak RG, Beroukhim R, Campbell PJ, Chin L, Dixon JR, Futreal PA, (2020). Disruption of chromatin folding domains by somatic genomic rearrangements in human cancer. Nat. Genet 52, 294–305. 10.1038/S41588-019-0564-Y. 32024999 PMC7058537

[41] Aguilera A, and García-Muse T (2013). Causes of genome instability. Annu. Rev. Genet 47, 1–32. 10.1146/ANNUREV-GENET-111212-133232. 23909437

[42] Bunting SF, and Nussenzweig A (2013). End-joining, translocations and cancer. Nat. Rev. Cancer 13, 443–454. 10.1038/nrc3537. 23760025 PMC5724777

[43] McVey M, and Lee SE (2008). MMEJ repair of double-strand breaks (director’s cut): deleted sequences and alternative endings. Trends Genet. 24, 529–538. 18809224 10.1016/j.tig.2008.08.007 PMC5303623

[44] Parker M, Mohankumar KM, Punchihewa C, Weinlich R, Dalton JD, Li Y, Lee R, Tatevossian RG, Phoenix TN, Thiruvenkatam R, (2014). C11orf95–RELA fusions drive oncogenic NF-kB signalling in ependymoma. Nature 506, 451–455. 10.1038/nature13109. 24553141 PMC4050669

[45] Mack SC, and Taylor MD (2017). Put away your microscopes: The ependymoma molecular era has begun. Curr. Opin. Oncol 29, 443–447. 10.1097/CCO.0000000000000411. 28885433 PMC6003664

[46] Rausch T, Jones DTW, Zapatka M, Stütz AM, Zichner T, Weischenfeldt J, Jäger N, Remke M, Shih D, Northcott PA, (2012). Genome sequencing of pediatric medulloblastoma links catastrophic DNA rearrangements with TP53 mutations. Cell 148, 59–71. 10.1016/j.cell.2011.12.013. 22265402 PMC3332216

[47] Notta F, Chan-Seng-Yue M, Lemire M, Li Y, Wilson GW, Connor AA, Denroche RE, Liang S-B, Brown AMK, Kim JC, (2016). A renewed model of pancreatic cancer evolution based on genomic rearrangement patterns. Nature 538, 378–382. 10.1038/nature19823. 27732578 PMC5446075

[48] Molenaar JJ, Koster J, Zwijnenburg DA, van Sluis P, Valentijn LJ, van der Ploeg I, Hamdi M, van Nes J, Westerman BA, van Arkel J, (2012). Sequencing of neuroblastoma identifies chromothripsis and defects in neuritogenesis genes. Nature 483, 589–593. 10.1038/nature10910. 22367537

[49] Cortés-Ciriano I, Lee JJ-K, Xi R, Jain D, Jung YL, Yang L, Gordenin D, Klimczak LJ, Zhang C-Z, Pellman DS, (2020). Comprehensive analysis of chromothripsis in 2,658 human cancers using whole-genome sequencing. Nat. Genet 52, 331–341. 32025003 10.1038/s41588-019-0576-7 PMC7058534

[50] Kim KH, and Roberts CWM (2014). Mechanisms by which SMARCB1 loss drives rhabdoid tumor growth. Cancer Genet. 207, 365–372. 10.1016/J.CANCERGEN.2014.04.004. 24853101 PMC4195815

[51] Shapiro JA, Gaonkar KS, Spielman SJ, Savonen CL, Bethell CJ, Jin R, Rathi KS, Zhu Y, Egolf LE, Farrow BK, (2023). Open-PBTA: The Open Pediatric Brain Tumor Atlas. Cell Genom. 3, 100340. 10.1016/J.XGEN.2023.100340. 37492101 PMC10363844

[52] Cer RZ, Donohue DE, Mudunuri US, Temiz NA, Loss MA, Starner NJ, Halusa GN, Volfovsky N, Yi M, Luke BT, (2013). Non-B DB v2.0: a database of predicted non-B DNA-forming motifs and its associated tools. Nucleic Acids Res. 41, D94–D100. 10.1093/NAR/GKS955. 23125372 PMC3531222

[53] Singh R, and Berger B (2021). Deciphering the species-level structure of topologically associating domains. Preprint at bioRxiv. 10.1101/2021.10.28.466333.

[54] Zhang J, Lee D, Dhiman V, Jiang P, Xu J, McGillivray P, Yang H, Liu J, Meyerson W, Clarke D, (2020). An integrative ENCODE resource for cancer genomics. Nat. Commun 11, 3696. 10.1038/S41467-020-14743-W. 32728046 PMC7391744

[55] Ng PC, and Henikoff S (2003). SIFT: predicting amino acid changes that affect protein function. Nucleic Acids Res. 31, 3812–3814. 10.1093/NAR/GKG509. 12824425 PMC168916

