# Supplementary material for: Somatic structural variation signatures in pediatric brain tumors
Source: Cell Rep. Author manuscript; Available in PMC 2023 Dec 24. (PMC10748741; doi:10.1016/j.celrep.2023.113276)
Supplement: 4 [file NIHMS1941933-supplement-4.pdf]

Cell Reports, Volume *42*

Supplemental information

Somatic structural variation signatures in pediatric brain tumors

Yang Yang and Lixing Yang

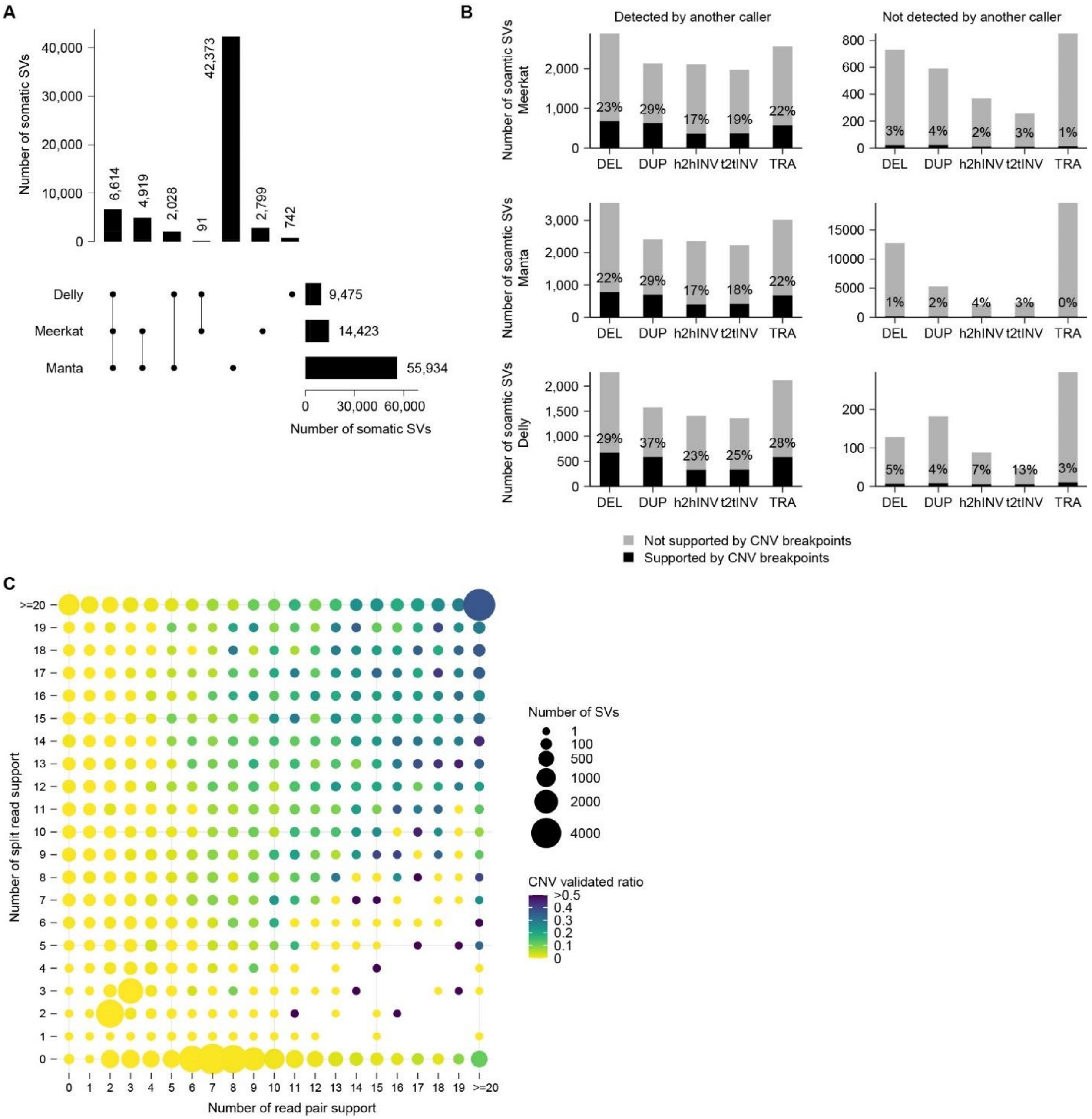

Figure S1 Quality control of somatic SVs called by Manta, Meerkat, and Delly, related to Figure 1 and STAR Methods. (A) UpSet plot of somatic SVs called by Meerkat, Manta, and Delly. The bars on the bottom right are the total SVs detected by the three tools respectively. The bars on the top show the number of SVs identified by one or more tools. The black dots under the bars indicate tools used. The numbers on the top and on the right side of the bars are numbers of SVs. (B) SVs called by different tools validated by CNVs. The left panels are high confidence SVs called by more than one tool, and the right panels are tool-specific low confidence SVs. The black and grey bars indicate SVs supported or not supported by CNV breakpoints, respectively. (C) CNV validation of Manta SVs. The x- and y-axes are the numbers of read pair and split-read

support for SVs detected by Manta, respectively. The size of each dot represents the number of SVs, and the color indicates the proportion of SVs validated by CNVs.

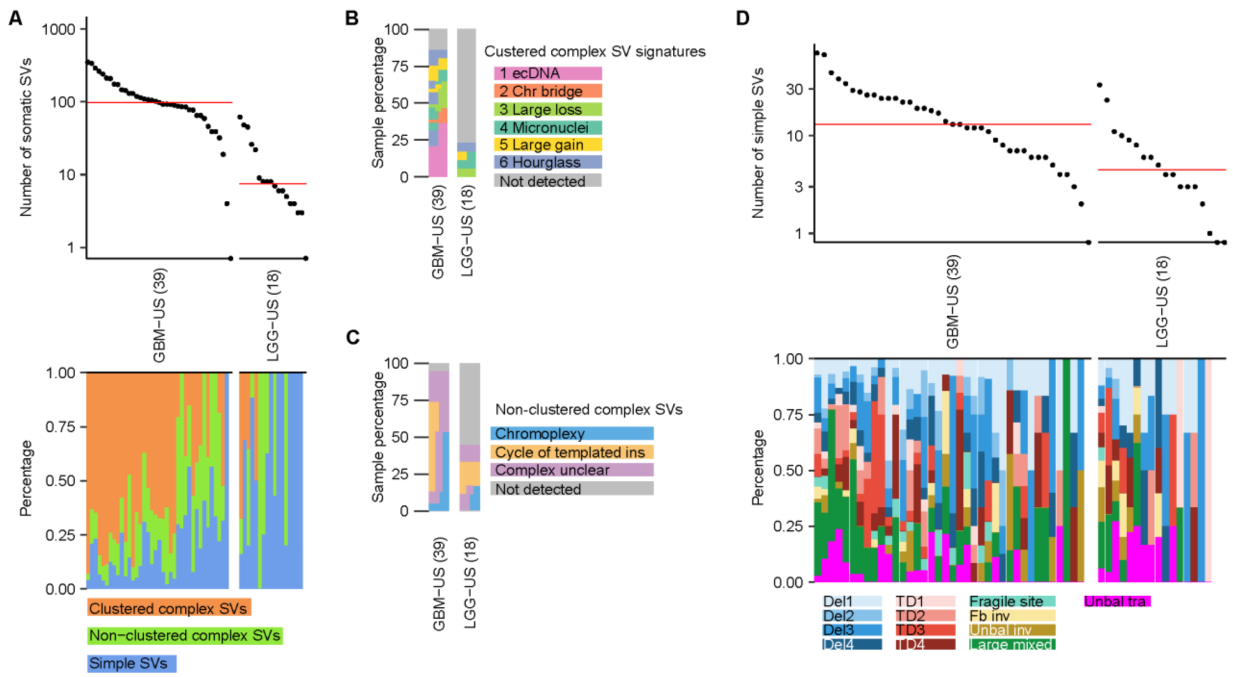

Figure S2 Somatic SVs in PCAWG adult brain tumors, related to Figures 1 and 2. (A) Frequencies of somatic SVs and percentages of different types of SVs in adult brain tumors. In the upper panel, each dot represents one adult brain tumor sample. Samples are grouped by tumor type and tumor types are sorted by median SV frequency (red lines). The numbers in parentheses are sample sizes for the corresponding tumor types. The bottom panel shows the percentages of clustered complex SVs, non-clustered complex SVs, and simple SVs in the corresponding samples on the top panel. GBM: glioblastoma multiforme. LGG: low-grade gliomas. (B) and (C) Percentages of clustered complex SV signatures and non-clustered complex SVs. Each vertical block represents one tumor type and each horizontal bar represents one sample. Samples are colored based on their SV signatures. Samples carry multiple signatures have multiple colors arranged horizontally. The height of each sample may differ across tumor types depending on sample sizes of the tumor types. (D) Frequencies of somatic simple SVs and percentages of different adult simple SV signatures in adult brain tumors. Samples are grouped by tumor type and tumor types are sorted by median SV frequency (red lines). The numbers in parentheses are sample sizes for the corresponding tumor types. The bottom panel shows the percentages of different adult simple SV signatures in the corresponding samples on the top panel.

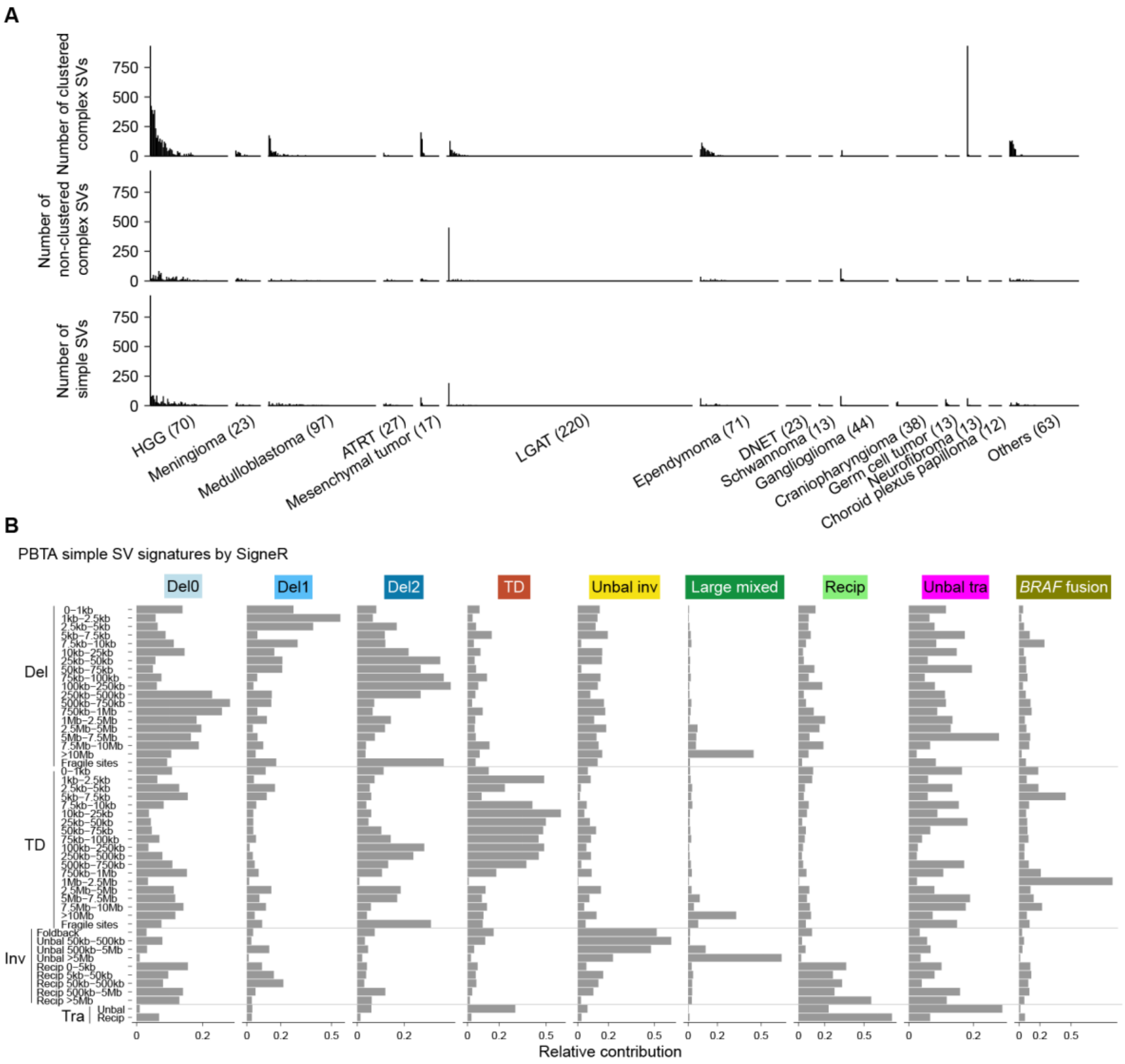

Figure S3 Frequencies of complex SVs, simple SVs, and simple SV signatures decomposed by signeR, related to Figures 1 and 2. (A) The three tracks present the number of clustered complex SVs, non-clustered complex SVs, and simple SVs, respectively. The tumor type ordering is consistent with Figure 1, and samples are sorted by the total number of SVs. The numbers in parentheses represent the sample sizes for the corresponding tumor types. (B) Nine simple SV signatures of 744 pediatric brain tumors. The four major SV categories and 49 subcategories of simple SVs are shown on the y-axis. The names of the nine simple SV signatures are displayed on the top. The relative contributions of SV subcategories to the corresponding signatures are shown on the x-axis.

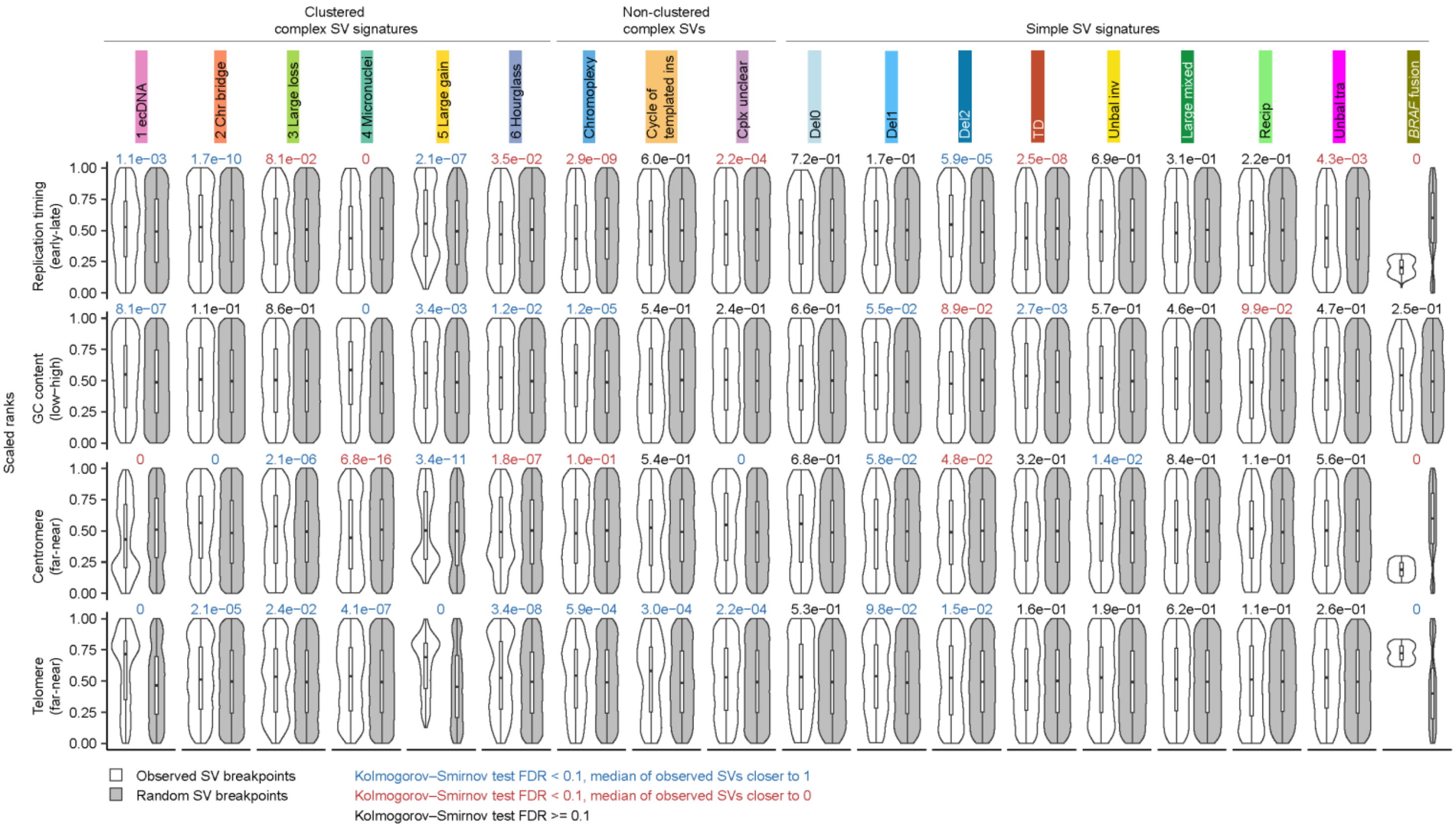

Figure S4 Comparisons of four genomic features between observed and randomly generated SVs, related to Figure 3. SV signatures and genomic features are listed in the x- and y-axes respectively. The violin and box plots display the distribution of scaled ranked genomic features. Colored numbers on top of each panel indicate the FDRs of the Kolmogorov-Smirnov test, as consistent with **Figure 3**.

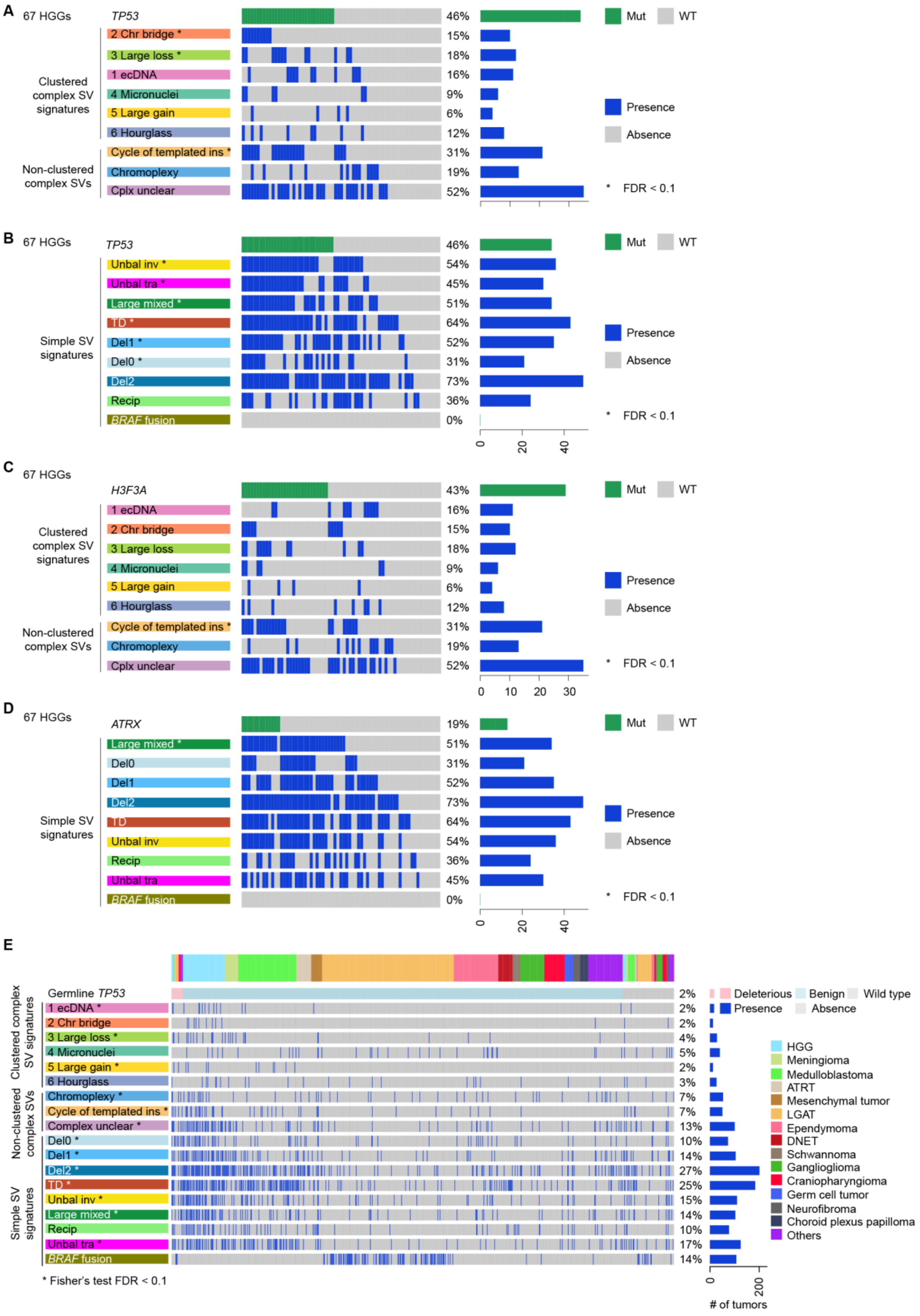
A
67 HGGs
TP53
46%
Mut
WT
2 Chr bridge *
15%
3 Large loss *
18%
1 ecDNA
16%
4 Micronuclei
9%
5 Large gain
6%
6 Hourglass
12%
Clustered complex SV signatures
Cycle of templated ins *
31%
Chromoplexy
19%
Cplx unclear
52%
Non-clustered complex SVs
Presence
Absence
* FDR < 0.1
B
67 HGGs
TP53
46%
Unbal inv *
54%
Unbal tra *
45%
Large mixed *
51%
TD *
64%
Del1 *
52%
Del0 *
31%
Del2
73%
Recip
36%
BRAF fusion
0%
Simple SV signatures
0
20
40
C
67 HGGs
H3F3A
43%
1 ecDNA
16%
2 Chr bridge
15%
3 Large loss
18%
4 Micronuclei
9%
5 Large gain
6%
6 Hourglass
12%
Cycle of templated ins *
31%
Chromoplexy
19%
Cplx unclear
52%
0
10
20
30
D
67 HGGs
ATRX
19%
Large mixed *
51%
Del0
31%
Del1
52%
Del2
73%
TD
64%
Unbal inv
54%
Recip
36%
Unbal tra
45%
BRAF fusion
0%
E
Germline TP53
2%
Deleterious
Benign
Wild type
Presence
Absence
1 ecDNA *
2%
2 Chr bridge
2%
3 Large loss *
4%
4 Micronuclei
5%
5 Large gain *
2%
6 Hourglass
3%
Clustered complex SV signatures
Chromoplexy *
7%
Cycle of templated ins *
7%
Complex unclear *
13%
Non-clustered complex SVs
Del0 *
10%
Del1 *
14%
Del2 *
27%
TD *
25%
Unbal inv *
15%
Large mixed *
14%
Recip
10%
Unbal tra *
17%
BRAF fusion
14%
Simple SV signatures
HGG
Meningioma
Medulloblastoma
ATRT
Mesenchymal tumor
LGAT
Ependymoma
DNET
Schwannoma
Ganglioglioma
Craniopharyngioma
Germ cell tumor
Neurofibroma
Choroid plexus papilloma
Others
* Fisher's test FDR < 0.1
0
200
# of tumors

Figure S5 Associations between SV signatures and mutations, related to Figure 3. (A) The associations between *TP53* mutations and complex SV signatures in 67 non-hypermutated HGGs. (B) The associations between *TP53* mutations and simple SV signatures in 67 non-hypermutated HGGs. (C) The associations between *H3F3A* mutations and complex SV signatures in 67 non-hypermutated HGGs. (D) The associations between *ATRX* mutations and simple SV signatures in 67 non-hypermutated HGGs. (E) The associations between SV signatures and germline *TP53* variants in 744 pediatric brain tumors. Tumor types are indicated by different colors on the top track. Protein-altering mutations are represented by green bars, while the presence of signatures is indicated by blue bars. The percentages reflect the proportions of samples carrying the corresponding mutations or signatures. The horizontal bars represent the frequencies of the corresponding mutations or signatures. The associations were assessed using Fisher's exact test, and the FDRs were adjusted using the Benjamini-Yekutieli method. Asterisks (*) denote statistically significant positive correlations (FDR < 0.1) between the tested genes (A-D) or deleterious germline *TP53* variants (E) and the corresponding signatures.

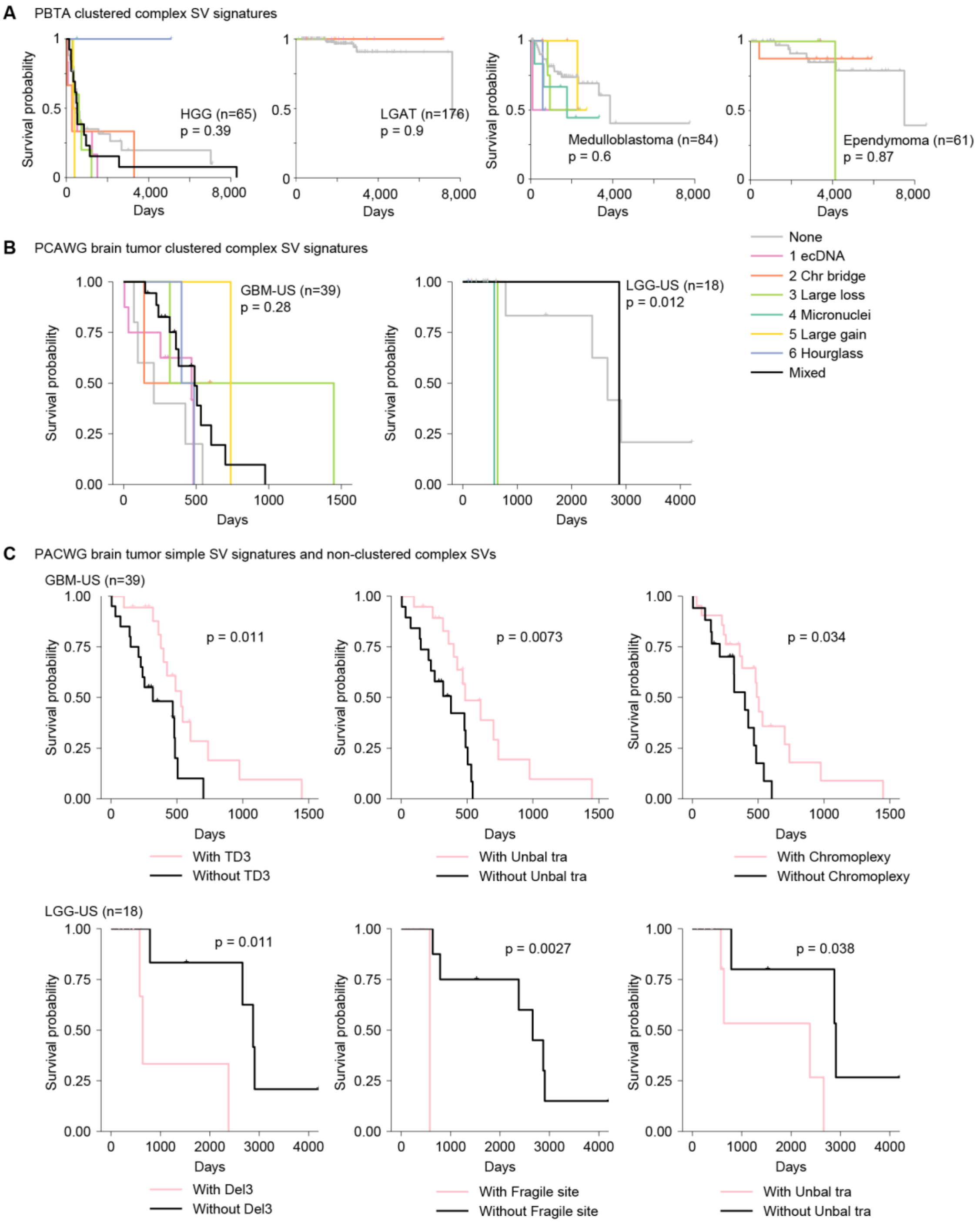

Figure S6 Kaplan–Meier survival curves for PBTA and PCAWG brain tumors, related to Figure 7. (A) Associations between clustered complex SV signatures and survival for PBTA four tumor types. The samples are colored by the clustered complex SV signatures. (B) Associations between clustered complex SV signatures and survival for PCAWG adult brain tumors. (C) Associations between simple SV signatures and survival for PCAWG adult brain tumors. Samples are stratified by the presence or absence of simple SV signatures. Samples are

colored by the clustered complex SV signatures (B and C). Log-rank test was used to calculate p-values. The sample sizes are provided in parentheses.

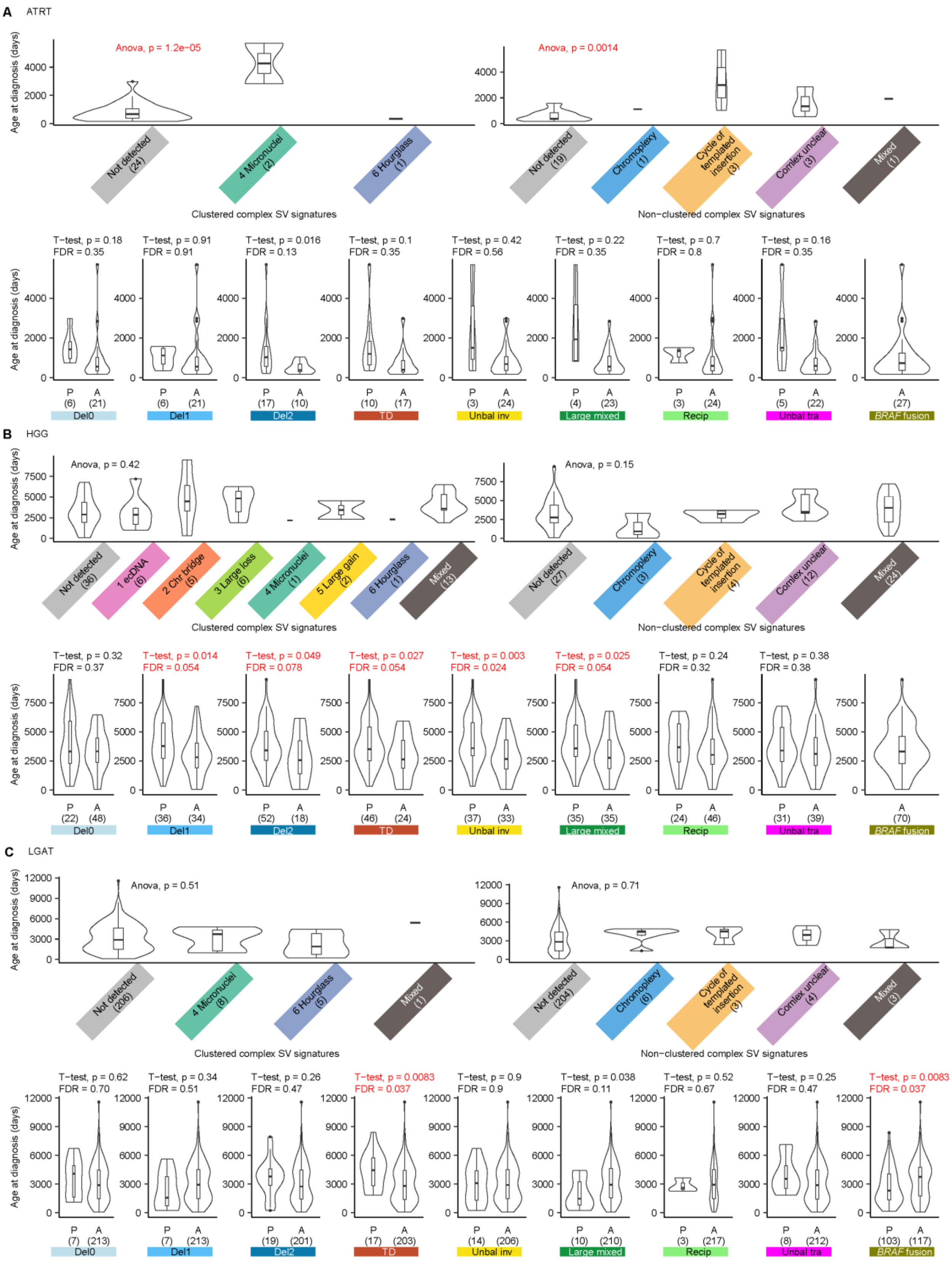

P: Presence of specific signature
A: Absence of specific signature

Figure S7 Associations between SV signatures and age of diagnosis, related to Figure 7 (A) Associations between SV signatures and age of diagnosis in ATRT. (B) Associations between SV signatures and age of diagnosis in HGG. (C) Associations between SV signatures and age of diagnosis in LGAT. Samples are stratified based on clustered complex SV signatures (upper left of each panel), non-clustered complex SVs (upper right of each panel), and simple SV signatures (bottom of each panel). The differences between groups were tested using ANOVA and Student's t test. The FDRs were corrected using the Benjamini-Yekutieli method. The sample sizes of each group are indicated in parentheses.

Sample_ID	Chr	Pos	SNP	Ref	Alt	Mutation_type	Tumor_type
BS_G9MQM1KK	chr17	7675145	.	CGGGTGCCGGGCGG	C	frameshift_variant	HGG
BS_9GSWPZ44	chr17	7676278	rs35117667	G	A	splice_region_variant&intron_variant	LGAT
BS_RXSBJ929	chr17	7675994	.	C	G	splice_region_variant&synonymous_variant	HGG
BS_5KFAEMQJ	chr17	7674298	.	A	G	splice_region_variant&intron_variant	Medulloblastoma
BS_80078QDG	chr17	7675994	rs55863639	C	A	splice_region_variant&synonymous_variant	HGG
BS_BMHYK93N	chr17	7676278	rs35117667	G	A	splice_region_variant&intron_variant	Other tumor
BS_16TBX6G5	chr17	7676023	.	A	C	missense_variant; deleterious(0.05)	LGAT
BS_XEVMEYFS	chr17	7673786	.	AG	A	frameshift_variant	Other tumor
BS_SWHQ8FDW	chr17	7675232	.	G	A	missense_variant; deleterious(0)	ATRT
BS_6Q9PMNWY	chr17	7676278	rs35117667	G	A	splice_region_variant&intron_variant	LGAT
BS_59FR1NC2	chr17	7675995	.	G	C	missense_variant&splice_region_variant; deleterious(0)	Other tumor
BS_HN8DE43A	chr17	7675128	.	T	A	missense_variant; deleterious(0)	Other tumor
BS_K14VJ1E3	chr17	7676278	rs35117667	G	A	splice_region_variant&intron_variant	Craniopharyngioma
BS_W8DA9AXT	chr17	7675151	.	C	T	missense_variant; deleterious(0.02)	Craniopharyngioma
BS_QMHND3QK	chr17	7676278	rs35117667	G	A	splice_region_variant&intron_variant	ATRT
BS_VA07453Z	chr17	7676278	rs35117667	G	A	splice_region_variant&intron_variant	LGAT
BS_9GJHMA3J	chr17	7674887	.	C	T	missense_variant; deleterious(0)	Ependymoma

Table S4 Deleterious germline TP53 variants, related to Figure 3 and STAR Methods.

**Sample_ID**	**Kids_first_participant_ID**	**Primary_site**	**Ependymoma_subtype**
BS_376Q9QKG	PT_1DY2TP9N	Spinal Cord- Lumbar/Thecal Sac	SP
BS_R8ZKDFWH	PT_YXZPWFVB	Spinal Cord- Cervical;Spinal Cord- Thoracic	SP
BS_9GJHMA3J	PT_MT27S7S4	Thalamus;Ventricles	ST Ventricles mixed
BS_0XR7GYAV	PT_XHYBZKCX	Spinal Cord- Cervical;Spinal Cord- Thoracic	SP
BS_83MSWR59	PT_99S5BPE3	Frontal Lobe;Temporal Lobe	ST
BS_9JVGXA2W	PT_KVFS3Z2Q	Spinal Cord- Lumbar/Thecal Sac	SP
BS_55N7KFHW	PT_S8E3281G	Spinal Cord- Cervical	SP
BS_A3EV6FXK	PT_1Z015H6V	Spine NOS	SP
BS_MVC1NCZX	PT_BWN251DF	Frontal Lobe;Parietal Lobe	ST
BS_VK46N40F	PT_QZN7FZER	Ventricles	Ventricles
BS_6FYTRKJJ	PT_625X34JX	Spinal Cord- Lumbar/Thecal Sac;Spinal Cord- Thoracic	SP
BS_H8E4K1DT	PT_46F28BFH	Occipital Lobe	ST
BS_6T87HSPX	PT_ZWY04DCR	Frontal Lobe	ST
BS_QSMFVHSB	PT_0WKX8Q5X	Spinal Cord- Lumbar/Thecal Sac	SP
BS_KB9GJDCS	PT_EB89G70N	Cerebellum/Posterior Fossa;Ventricles	PF Ventricles mixed
BS_ST3Z2B9B	PT_2SCVQ2N6	Parietal Lobe;Ventricles	ST Ventricles mixed
BS_01DQH017	PT_CYVVA9AB	Occipital Lobe	ST
BS_B4P1NP87	PT_3VCS1PPF	Spinal Cord- Cervical;Spinal Cord- Lumbar/Thecal Sac;Spinal Cord- Thoracic	SP
BS_5V2XCW17	PT_3KWSRWZN	Ventricles	Ventricles
BS_R7YMKNJN	PT_RM5S859Q	Occipital Lobe	ST
BS_2P7MJGM7	PT_EG7PH32E	Spinal Cord- Lumbar/Thecal Sac	SP
BS_XBE002WV	PT_D3AN5M5W	Ventricles	Ventricles
BS_EJ1H9PZY	PT_DNAJYFZT	Occipital Lobe	ST
BS_NWYBD9CA	PT_82A9SDRN	Parietal Lobe;Ventricles	ST Ventricles mixed
BS_ZBWQG7VR	PT_PKP1250P	Frontal Lobe	ST
BS_ZS1QRMXS	PT_8RB7TPS2	Temporal Lobe	ST
BS_MAVSJH2Y	PT_JN3EN2HR	Brain Stem-Pons;Cerebellum/Posterior Fossa;Cranial Nerves NOS	PF Other mixed
BS_AVS4DSZW	PT_N9W4GT6D	Spinal Cord- Lumbar/Thecal Sac	SP
BS_T1SNKF50	PT_CYBYN18G	Parietal Lobe	ST

BS_W74D279Q	PT_JP1FDKN9	Frontal Lobe	ST
BS_1AZ8YJSH	PT_R6WWH1QX	Frontal Lobe;Parietal Lobe	ST
BS_ZZJF26C4	PT_ZA95JQEB	Ventricles	Ventricles
BS_5D24XV4T	PT_164RNWTT	Cerebellum/Posterior Fossa	PF
BS_9E632VPJ	PT_GAM3E6M3	Frontal Lobe;Parietal Lobe	ST
BS_H8MAKDRB	PT_GH27VJBD	Brain Stem-Midbrain/Tectum;Cerebellum/Posterior Fossa	PF Other mixed
BS_AGD2ATY1	PT_ZZRBX5JT	Ventricles	Ventricles
BS_MFTAVQ5C	PT_T4VKB29K	Cerebellum/Posterior Fossa	PF
BS_007JTNB8	PT_1MW98VR1	Cerebellum/Posterior Fossa	PF
BS_BBCM52QE	PT_23K3YS1P	Parietal Lobe	ST
BS_6Z4WHYDG	PT_8SDJXNFR	Occipital Lobe;Parietal Lobe	ST
BS_HBAKYKQM	PT_C94VR1Y2	Spinal Cord- Lumbar/Thecal Sac	SP
BS_BBHFKBE7	PT_VE0Q731Y	Cerebellum/Posterior Fossa	PF
BS_K6A9Z04J	PT_S4H6KA09	Frontal Lobe	ST
BS_JC2187NA	PT_2CJ31SBX	Cerebellum/Posterior Fossa	PF
BS_6ZP2KRPW	PT_Z4GS3ZQQ	Frontal Lobe	ST
BS_W5P7SPDH	PT_NPETR8RY	Ventricles	Ventricles
BS_GMQZNQH8	PT_V0X73HEQ	Cerebellum/Posterior Fossa;Spinal Cord- Cervical	PF SP mixed
BS_QBZDQX7A	PT_EZW3S4F1	Cerebellum/Posterior Fossa	PF
BS_5ZRZC3ZM	PT_WKNKNYHH	Cerebellum/Posterior Fossa	PF
BS_Y3472DYJ	PT_N4PR21ME	Ventricles	Ventricles
BS_0W8AWY10	PT_06H29FCG	Cerebellum/Posterior Fossa	PF
BS_62XRDTM6	PT_K2YDNZDA	Ventricles	Ventricles
BS_J33YVP27	PT_ZD45GXZ0	Cerebellum/Posterior Fossa	PF
BS_F5V8CWXT	PT_3PK66TJY	Cerebellum/Posterior Fossa	PF
BS_6NPSZJ4C	PT_NYBH025T	Cerebellum/Posterior Fossa	PF
BS_G16NM5WJ	PT_7E3V3JFX	Cerebellum/Posterior Fossa	PF
BS_BSM3ZHW4	PT_6TMT65YA	Cerebellum/Posterior Fossa	PF
BS_HQFNQHVW	PT_0NY38X3W	Cerebellum/Posterior Fossa;Ventricles	PF Ventricles mixed
BS_MGBEMNYR	PT_ECMZCNTF	Frontal Lobe	ST
BS_75VHXMZN	PT_3R0P995B	Cerebellum/Posterior Fossa;Suprasellar/Hypothalamic/Pituitary	PF ST mixed
BS_4PAE19ZC	PT_67R1AF5R	Ventricles	Ventricles
BS_99PPRCW4	PT_BNARR0N6	Cerebellum/Posterior Fossa	PF

BS_EFBBP9H3	PT_9AW9EWHA	Brain Stem-Pons;Cerebellum/Posterior Fossa	PF Other mixed
BS_XMNBSBGN	PT_H876VB6R	Cerebellum/Posterior Fossa	PF
BS_7RQCH5Y7	PT_V3Q78E6F	Ventricles	Ventricles
BS_9PQPZPGM	PT_N9KYV4WB	Cerebellum/Posterior Fossa	PF
BS_9ZFXXJPK	PT_VBVZ2NAR	Brain Stem- Midbrain/Tectum	Other
BS_CFPWSZB5	PT_6BX46NVR	Other locations NOS	Other
BS_2E81A3FT	PT_ZZWYKZZ8	Cerebellum/Posterior Fossa;Ventricles	PF Ventricles mixed
BS_R82MYPKB	PT_FHDZNN40	Cerebellum/Posterior Fossa	PF
BS_FVPMPMRJ	PT_W17NV5YG	Parietal Lobe	ST

Table S5 Ependymoma subtypes, related to Figure 6.
